# A dorsal CA2 to ventral CA1 circuit contributes to oxytocinergic modulation of long-term social recognition memory

**DOI:** 10.1186/s12929-022-00834-x

**Published:** 2022-07-10

**Authors:** Tsung-Chih Tsai, Yi-Syuan Fang, Yu-Chieh Hung, Ling-Chien Hung, Kuei-Sen Hsu

**Affiliations:** 1 Institute of Basic Medical Sciences, College of Medicine, National Cheng Kung University, Tainan, 70101 Taiwan; 2 Department of Pharmacology, College of Medicine, National Cheng Kung University, No. 1, University Rd., Tainan, 70101 Taiwan; 3 Division of Neurology, Department of Internal Medicine, Ditmanson Medical Foundation Chia-Yi Christian Hospital, Chiayi, 60002 Taiwan

**Keywords:** Oxytocin, Oxytocin receptor, Social recognition memory, CA2, Hippocampus, Paraventricular nucleus

## Abstract

**Background:**

Social recognition memory (SRM) is the ability to distinguish familiar from novel conspecifics and is crucial for survival and reproductive success across social species. We previously reported that oxytocin (OXT) receptor (OXTR) signaling in the CA2/CA3a of dorsal hippocampus is essential to promote the persistence of long-term SRM, yet how the endogenous OXT system influences CA2 outputs to regulate long-term SRM formation remains unclear.

**Methods:**

To achieve a selective deletion of CA2 OXTRs, we crossed *Amigo2*-Cre mice with *Oxtr*-floxed mice to generate CA2-specific *Oxtr* conditional knockout (*Oxtr*^−/−^) mice. A three-chamber paradigm test was used for studying SRM in mice. Chemogenetic and optogenetic targeting strategies were employed to manipulate neuronal activity.

**Results:**

We show that selective ablation of *Oxtr* in the CA2 suffices to impair the persistence of long-term SRM but has no effect on sociability and social novelty preference in the three-chamber paradigm test. We find that cell-type specific activation of OXT neurons within the hypothalamic paraventricular nucleus enhances long-term SRM and this enhancement is blocked by local application of OXTR antagonist L-368,899 into dorsal hippocampal CA2 (dCA2) region. In addition, chemogenetic neuronal silencing in dCA2 demonstrated that neuronal activity is essential for forming long-term SRM. Moreover, chemogenetic terminal-specific inactivation reveals a crucial role for dCA2 outputs to ventral CA1 (vCA1), but not dorsal lateral septum, in long-term SRM. Finally, targeted activation of the dCA2-to-vCA1 circuit effectively ameliorates long-term SRM deficit observed in *Oxtr*^−/−^ mice.

**Conclusions:**

These findings highlight the importance of hippocampal CA2 OXTR signaling in governing the persistence of long-term SRM and identify a hippocampal circuit linking dCA2 to vCA1 necessary for controlling long-term SRM formation.

**Supplementary Information:**

The online version contains supplementary material available at 10.1186/s12929-022-00834-x.

## Background

Social recognition memory (SRM) refers to the ability of animals and humans to recognize and memorize familiar conspecifics and this ability is critical for maintaining appropriate social relationships [1–3]. In animals, SRM is essential for forming social groups and establishing hierarchies and social and affective ties [4, 5]. The development of SRM in humans and primates relies heavily on the use of visual and auditory cues, whereas other mammals such as rodents rely their SRM processing on multisensory integration of olfactory, auditory and somatosensory cues [4, 6]. Previous studies on the neural basis of SRM formation in rodents have implicated multiple brain regions, including the hippocampus, amygdala and anterior cingulate cortex [7–13]. The hippocampus in particular has been firmly established as a key region in SRM formation. Indeed, bilateral lesions of the hippocampus with ibotenic acid result in an impaired SRM in mice [7]. Thus, the hippocampus is an attractive brain region for the study of neurobiological mechanisms underlying SRM. Within the hippocampus, distinct subregions may contribute differentially to encode and retrieve mnemonic information through their differential connections. Of these subregions, recent studies have shed light on the importance of dorsal CA2 (dCA2) in SRM processing. For example, excitatory lesion or genetically targeted inactivation of dCA2 pyramidal neurons was found to selectively impair SRM with no change in sociability [9, 13, 14]. Additional studies have shown that dCA2 contributes to SRM by providing strong excitatory inputs to ventral CA1 (vCA1) that contains a particular subset of neurons implicated in SRM storage [13, 15]. Furthermore, by interfering with different phases of social memory tasks, Meira et al. [13] showed that dCA2 is necessary for all phases of SRM, including encoding, consolidation and recall. Despite these observations, much less is known about the neuromodulatory mechanisms engaged CA2 circuits in regulating SRM formation.

The hypothalamic neuropeptide oxytocin (OXT) promotes various aspects of social behaviors [16, 17]. OXT is mainly synthesized in neurons of the paraventricular nucleus (PVN) and supraoptic nucleus. While the vast majority of OXT neurons project to the posterior pituitary, they also sent axon collaterals to various forebrain regions that express OXT receptors (OXTRs), including the central nucleus of the amygdala, lateral septum (LS), nucleus accumbens (NAc), medial prefrontal cortex and hippocampus [18–20]. We have recently shown that OXTRs are prominently expressed in hippocampal CA2 and CA3 pyramidal neurons of mice and conditional deletion of CA2/CA3a *Oxtr* impairs the persistence of long-term SRM [12]. Accordingly, viral-mediated deletion of *Oxtr* in anterior CA2/CA3 neurons has been shown to impair discrimination of social, but not non-social, stimuli in mice [21]. Nonetheless, silencing dCA3 alone had no significant effect on SRM [22]. These findings highlight the importance of CA2 OXTR signaling in modulating the persistence of SRM. Nevertheless, it remains unclear how the endogenous OXT system and CA2 outputs regulate long-term SRM formation. Here, to specifically examine the functional and behavioral relevance of CA2 OXTRs, we used a CA2-selective *Amigo2*-Cre mouse line to obtain selective deletion of *Oxtr* in CA2 neurons [9]. To bridge of our understanding of endogenous OXTergic neuromodulation, we determined the impact of selective chemogenetic activation of PVN OXT neurons on long-term SRM formation. Finally, we explored CA2 outputs that underpin long-term SRM formation using circuit-based chemogenetic approaches. We report that the endogenous OXT system strongly promotes the persistence of long-term SRM by acting through the dCA2-to-vCA1 circuit.

## Materials and methods

### Animals

All experimental procedures were approved by the Institutional Animal Care and Use Committee (IACUC) at the National Cheng Kung University (authorization #107023) and conformed to the National Institutes of Health Guide for the Care and Use of Laboratory Animals. Homozygous *Oxtr*-floxed (*Oxtr*^J/J^; donating investigator: Dr. W. Scott Young III) and heterozygous *Amigo2*-Cre transgenic mice (donating investigator: Dr. Steven A. Siegelbaum) were obtained from The Jackson Laboratory. We crossed *Amigo2*-Cre mice with *Oxtr*^J/J^ mice to generate CA2-specific *Oxtr* conditional knockout (*Oxtr*^−/−^) mice, which are maintained on the C57BL/6 genetic background. *Oxtr*^J/J^ mice were used as the wild-type (WT) littermates for comparison with homozygous *Oxtr*^−/−^ mice. The heterozygous OXTR-Venus knock-in (*Oxtr*^Venus−Neo/+^) mice were obtained from Dr. Katsuhiko Nishimori and characterized as described previously [23]. Genotyping was performed by the polymerase chain reaction (PCR)-based method using genomic DNA isolated from tail samples as previously described [24]. All mice were housed in groups of three with same-sex littermates, kept in a 12 h light/12 h dark cycle and had ad libitum access to food and water, in the humidity- and temperature-controlled (25 ± 1 °C) specific pathogen-free animal facility. Experiments were done with adult mice starting at 10–12 weeks old and all behavioral testing were performed in the light phase. We used only male mice because of the sex bias in the three-chamber paradigm test [25, 26], in which female mice show lower social investigatory behavior compared to male mice throughout test. All experiments were conducted blinded to the genotype and experimental designs.

### Recombinant adeno-associated virus (AAV) vector production

DNA plasmids encoding pAAV-hSyn-DIO-mCherry (Addgene, plasmid #50459), pAAV-hSyn-DIO-hM3D(Gq)-mCherry (Addgene, plasmid #44361), pAAV-hSyn-DIO-hM4D(Gi)-mCherry (Addgene, plasmid #44362) and pAAV-mOXT-hM3D(Gq)-mCherry (Addgene, plasmid #70717) were purchased from Addgene. Plasmid DNA was amplified, purified and collected with the QIAGEN plasmid maxiprep kit following manufacturer’s instructions. The purified plasmids were mixed into CaCl_2_ solution with the DNA plasmid coding AAV5 and co-transfected into HEK293GP cells using the calcium phosphate co-precipitation method as described previously [12, 24]. Transfected cells were harvested 72 h after transfection and viruses were purified using the AAV Purification Mega Kit (Cell Biolabs, Inc.). Viral titers were 5 × 10^12^ particles/ml and stored in aliquots at − 80 °C until use.

### Stereotaxic viral injections and chemogenetic manipulations

Under isoflurane (5% induction and 2% maintenance; Attane) anesthesia, concentrated virus-stock solution was stereotaxically injected into the targeted sites (0.5 μl per site at 0.25 μl/min) by using a Hamilton syringe with a 34-gauge blunted-tip needle. Buprenorphine (0.1 mg/kg) was subcutaneously delivered prior to the surgery to reduce discomfort. Body temperature was maintained at 37 °C via a heating pad. For chemogenetic activation of PVN OXT neurons, we bilaterally injected AAV_5_-mOXT-hM3D(Gq)-mCherry into the PVN of the hypothalamus according to the following injection coordinates: anteroposterior (AP) − 0.8 mm, mediolateral (ML) ± 0.2 mm and dorsoventral (DV) − 5 mm. For chemogenetic manipulation of dCA2, we bilaterally injected AAV_5_-hSyn-DIO-mCherry, AAV_5_-hSyn-DIO-hM4D(Gi)-mCherry or AAV_5_-hSyn-DIO-hM3D(Gq)-mCherry into the hippocampus of *Amigo2*-Cre or *Oxtr*^−/−^ mice according to the following injection coordinates: AP − 2.3 mm, ML ± 2.7 mm and DV − 2.0 mm. For silencing of dCA2-to-vCA1 projections, AAV_5_-hSyn-DIO-hM4D(Gi)-mCherry was bilaterally injected into dCA2 and mice were bilaterally implanted with 26 gauge cannula guides (RWD Life Science Co., Ltd.) aimed at vCA1 (AP − 3.0 mm, ML ± 2.5 mm and DV − 2.0 mm) to deliver vehicle (PBS, 0.5 μl) or clozapine-*N*-oxide (CNO, 4 mM in PBS, 0.5 μl; Sigma-Aldrich). For silencing of dCA2-to-dorsal LS (dLS) projections, AAV_5_-hSyn-DIO-hM4D(Gi)-mCherry was bilaterally injected into dCA2 and mice were bilaterally implanted with 26 gauge cannula guides aimed at dLS (AP + 0.2 mm, ML ± 0.4 mm and DV − 2.5 mm) to deliver vehicle (PBS, 0.5 μl) or CNO (4 mM in PBS, 0.5 μl). Dummy cannulas were inserted into guide cannulas and secured to skull with dental cement. All injections were performed on 10–12 weeks old male mice and were followed by a 3-week viral incubation period before the start of the behavioral testing. For chemogenetic activation of PVN OXT projection to dCA2, CNO (4 mM in PBS, 0.5 μl) was delivered locally through cannulas implanted bilaterally into dCA2 20 min before performing the three-chamber paradigm test. To better characterize the involvement of locally released OXT and subsequent activation of OXTR, L-368,899 (12 μg in PBS, 0.5 μl, Tocris Bioscience) was administrated bilaterally into the dCA2 10 min before CNO injection. Drug dose of L-368,899 was selected on the basis of published study [21]. For chemogenetic silencing of dCA2 neurons, *Amigo2*-Cre mice infected in dCA2 with AAV_5_-hSyn-DIO-hM4D(Gi)-mCherry or control AAV_5_-hSyn-DIO-mCherry were injected intraperitoneally with vehicle (5% DMSO in PBS) or CNO (5 mg/kg in 5% DMSO) 30 min before performing the three-chamber paradigm test. Dose of CNO was selected on the basis of published studies [27, 28]. After behavioral testing, brains were dissected and serial slices were imaged to verify correct viral expression and implant placement location.

### Fluorescent in situ hybridization

Fluorescent in situ hybridization (FISH) analysis was performed using RNAscope^®^ Multiplex Fluorescent Reagent Kit 2.0 manufacturer’s instructions (Advanced Cell Diagnostics; RRID: SCR_012481) as previously described [12, 24]. Coronal brain slices (16 μm) were fixed in 4% paraformaldehyde (PFA; Sigma-Aldrich) for 15 min and dehydrated in a 50%, 70% and 100% ethanol series for 5 min each. Sections were subjected to reagent Pretreat 3 at 25 °C for 30 min and then hybridized with probes at 40 °C for 2 h in a humidified oven (ACD HybEZ™ Hybridization System). The *Oxtr*-O1 probe (Cat# 454011) and the *Amigo2* probe (Cat# 504478) were used to target *Oxtr* mRNA and *Amigo2* mRNA, respectively. After hybridization, brain sections were sequentially applied with a series of probe signal amplification steps, washed with ACD Wash Buffer 2 times, 2 min each wash, and then mounted with VECTASHIELD antifade mounting medium (Vector Laboratories) containing 4′,6-diamidino-2-phenylindole (DAPI, 1:5000; Cat# D9542; Sigma-Aldrich) for staining DNA.

### Histology and quantification

Tissue preparation and histological analysis was performed as described previously [12]. Mice were deeply anesthetized with 5% isoflurane and perfused transcardially with cold (4 °C) PBS, followed by 4% PFA in 0.1 M PBS, pH 7.4. After the perfusion, brains were removed and fixed in 4% PFA for 24 h at 4 °C and then immersed for at least 48 h in the solution containing 30% sucrose at 4 °C before sectioning. Coronal brain slices containing the hippocampus were sectioned (20 μm thickness), washed with 0.3% Triton X-100, and then incubated with blocking solution containing 3% goat serum in PBS. For quantitative analysis of neuron numbers with Nissl staining, sections were mounted directly on gelatin-coated glass slides and dried. The slides were stained with 1.0% cresyl violet, dehydrated through a series of ethanol, cleared and coverslipped with Permount (Thermo Fisher Scientific). Nissl staining within the CA2 region was quantified in images from about 1.5 to 2.5 mm posterior to Bregma every sixth coronal section captured at 200× magnification and digitized with an Olympus BX51 microscope equipped with Olympus DP70 digital camera. All images were imported into NIH ImageJ software (RRID:SCR_001935) for analysis.

### Immunohistochemistry

Immunofluorescence staining was performed as described previously [12, 24]. In brief, *Oxtr*^Venus−Neo/+^ mice were deeply anesthetized with 5% isoflurane and perfused transcardially with 4 °C PBS, followed by 4% PFA in 0.1 M PBS, pH 7.4. After the perfusion, brains were removed and fixed in 4% PFA for 24 h at 4 °C and then transferred to the solution containing 30% sucrose that immersed in 4 °C for at least 48 h before slicing. Coronal slices were sectioned to a 40 μm thickness, washed with 0.3% Triton X-100, and then incubated for blocking with solution containing 3% goat serum in PBS for 30 min. After blocking, the sections were incubated with primary antibody against striatum-enriched protein-tyrosine phosphatase (STEP; also known as PTPN5) (1:500, Cell Signaling Technology, Cat #4376; RRID: AB_1904101). Finally, sections were washed with TBS-T (10 mM Tris–HCl, 150 mM NaCl and 0.025% Tween 20; pH 7.4) and then incubated with Alexa Fluor 568 antibody (1:500, Life Technologies) secondary antibody for 2 h at room temperature in blocking solution. The immunostained sections were collected on separate gelatin-subbed glass slides, rinsed extensively in PBS, and mounted with ProLong Gold Antifade Reagent (Invitrogen). Post hoc tissue images were acquired using an Olympus FluoView FV3000 confocal laser scanning microscope. All images were imported into NIH ImageJ software for analysis.

### Three-chamber paradigm test

The three-chamber paradigm test was conducted as previously described [12]. The apparatus was a custom-built rectangular box (60 cm × 40 cm × 22 cm) divided into three chambers made from clear polycarbonate, with openings (10 cm width × 5 cm height) that allow access into each chamber. The assay consisted of three trials in a dimly lit room (~ 10–15 lx white light). The subject mouse was initially placed into the box and allowed to freely explore all three chambers for 10 min for habituation. After habituation phase, a juvenile male mouse (stimulus, 3–5 weeks old), which had no previous contact with the subject mice, was placed in a wire cage of left or right chamber (systemically alternated) and an empty wire cage was placed in the other side chamber. The subject mouse was placed in the middle chamber, and then the mouse was allowed to freely explore all three chambers for 5 min (sociability test). The time that the test subject spent in sniffing at the wire cage containing the juvenile stimulus mouse or the empty wire cage was measured. At the end of the sociability test, the subject mouse was gently guided to the center chamber while the empty wire cage was replaced with a novel unfamiliar male juvenile mouse (novel 1). The subject mouse again freely explored all three chambers for 5 min to quantify social preference for a novel stranger mouse (social novelty preference test). The subject mouse had a free choice between the first, already-investigated mouse (familiar) and the novel unfamiliar mouse. The time that the test subject spent in sniffing at the wire cage containing a familiar mouse or a novel mouse was measured. After social novelty test, mice were returned to their home cages. On day 2 or day 8, 1-day or 7-day long-term SRM was examined in which the familiar mouse was placed in the left or right chamber, and a novel unfamiliar male juvenile mouse (novel 2) was placed in the cage of other chamber. The subject mouse again freely explored all three chambers for 5 min. The subject mouse had a free choice between the familiar mouse and the novel unfamiliar mouse for 5 min. The time that the test subject spent in sniffing at the wire cage containing a familiar mouse or a novel mouse was measured. The behavior of the animals was videotaped and analyzed using the EthoVision XT video tracking system (Noldus; RRID: SCR_000441). The discrimination index for assessing sociability was calculated as [(time of sniffing the stimulus object − time of sniffing the empty cage)/(time of sniffing in both stimulus object and empty cage)]. The discrimination index for assessing social memory was calculated as [(time of sniffing the novel object − time of sniffing the familiar object)/(time of sniffing the objects in both novel and familiar object)].

### Experimental designs

During the experiment, we did not observe animal deaths due to experimental manipulations. In experiment 1, to examine the expression of OXTRs in hippocampal dCA2 subregion, we used transgenic *Oxtr*^Venus−Neo/+^ mice. We used *Oxtr*^J/J^ and *Oxtr*^−/−^ mice for histological analysis. In experiment 2, to determine the impact of CA2 OXTR deletion on SRM persistence, we used *Oxtr*^J/J^ and *Oxtr*^−/−^ mice in the three-chamber paradigm test. In experiment 3, to determine if the endogenous OXT system participates in regulating the formation of long-term SRM, AAV_5_-mOXT-hM3D(Gq)-mCherry-treated *Oxtr*^J/J^ mice were subjected to bilateral injection of either vehicle or CNO into dCA2 and then subjected to the three-chamber paradigm test. In experiment 4, to confirm the involvement of locally released OXT and subsequent activation of OXTR-mediated signaling in dCA2 during SRM encoding, AAV_5_-mOXT-hM3D(Gq)-mCherry/CNO-treated *Oxtr*^J/J^ mice were subjected to bilateral injection of either vehicle or L-368,899 into dCA2 and then subjected to the three-chamber paradigm test. In experiment 5, to probe causality between dCA2 activity and the formation of long-term SRM, *Amigo2*-Cre mice were infected with AAV_5_-hSyn-DIO-mCherry or AAV_5_-hSyn-DIO-hM4D(Gi)-mCherry in dCA2 and then subjected to the three-chamber paradigm test 30 min after intraperitoneal CNO injection. In experiment 6, to interrogate the role of dCA2-to-vCA1 projections in long-term SRM, *Amigo2*-Cre mice were infected with AAV_5_-hSyn-DIO-hM4D(Gi)-mCherry in dCA2 and then subjected to the three-chamber paradigm test after bilateral injection of either vehicle or CNO into vCA1. In experiment 7, to interrogate the role of dCA2-to-dLS projections in long-term SRM, *Amigo2*-Cre mice were infected with AAV_5_-hSyn-DIO-hM4D(Gi)-mCherry in dCA2 and then subjected to the three-chamber paradigm test after bilateral injection of either vehicle or CNO into dLS. In experiment 8, to investigate whether activation of dCA2-to-vCA1 projections can ameliorates long-term SRM deficit in *Oxtr*^−/−^ mice, *Oxtr*^−/−^ mice were infected with AAV_5_-hSyn-DIO-mCherry or AAV_5_-hSyn-DIO-hM3D(Gq)-mCherry in dCA2 and then subjected to the three-chamber paradigm test after bilateral injection of CNO into vCA1.

### Statistical analysis

No statistical methods were used to predetermine sample size, but the number of animals used in each experiment was chosen on the basis of previous studies in our laboratory [12, 24]. No specific randomization method was used in allocating an animal into a particular experimental conditions reported in this study. The data were expressed as mean ± SEM and statistical comparisons were conducted using Prism 6 software (GraphPad). Two-tailed paired Student’s *t*-test was used for within-group comparison and the unpaired Student’s *t*-test was used for comparison between two independent groups. Number of animals used is indicated by *n*. Probability values of *P* < 0.05 were considered to represent significant differences. The statistical results are described in Additional file 1.

## Results

### Conditional deletion of *Oxtr* from CA2 excitatory neurons

**Fig. 1:**
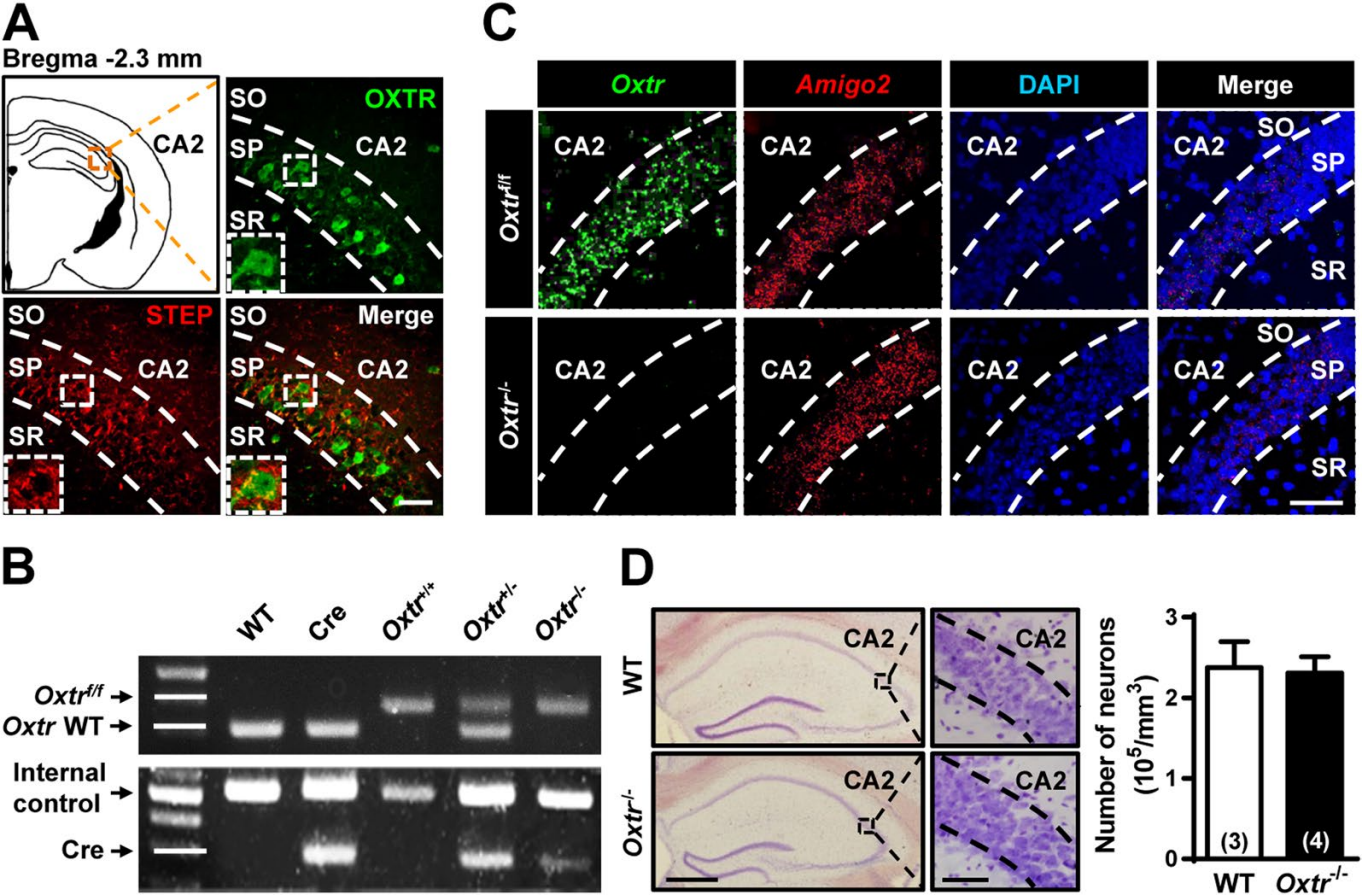
Deletion of *Oxtr* in CA2 excitatory neurons of the mouse hippocampus. **A** Doubled-labeled confocal immunofluorescent images showing the colocalization of OXTRs (green) and STEP (red) in dCA2 of the mouse hippocampus. The inserts represent high magnification of the boxed area. Scale bar: 50 μm. *SO* stratum oriens, *SP* stratum pyramidale, *SR* stratum radiatum. **B** PCR screening of tail-derived genomic DNA for selection of *Oxtr*^−/−^ mice. **C** Dual-probe FISH images showing the expression of *Oxtr* mRNA and *Amigo2* mRNA in dCA2 of WT and *Oxtr*^−/−^ mice (counterstained with DAPI, blue). Scale bar, 50 μm. Data was replicated in 4 mice. **D** Representative images with cresyl violet staining of dCA2 showing that the number of pyramidal neurons was not affected by targeted deletion of *Oxtr* compared with age-matched WT mice. Group data showing the summary results from 3–4 mice of each group at 12 weeks old. Scale bars: left, 500 μm; right, 50 μm. The total number of animal examined is indicated by *n* in parenthesis. Error bars represent the SEM

To first ensure the expression of OXTRs in hippocampal dCA2 subregion, we performed immunohistochemical staining using a transgenic *Oxtr*^Venus−Neo/+^ mouse line that expresses the Venus variant of yellow fluorescent protein in OXTR-expressing cells. Consistent with previous reports [23, 24], we observed strong expression of Venus-labeled cells in dCA2 stratum pyramidale of *Oxtr*^Venus−Neo/+^ mice. As illustrated by a single atlas plate schematic (bregna − 2.3 mm), double-labeling revealed co-localized expression of OXTR and putative CA2 marker STEP (Fig. 1A), indicating that OXTRs are indeed expressed in CA2 pyramidal neurons [29, 30]. To gain insight into the functional and behavioral relevance of OXTRs in CA2 pyramidal neurons, we took advantage of the Cre-loxP recombination approach to conditionally delete *Oxtr* from CA2 excitatory neurons by crossing *Oxtr*^f/f^ mice with *Amigo2*-Cre mice, in which Cre expression is largely restricted to CA2 pyramidal neurons [9, 31]. PCR screening of mouse genomic tail DNA confirmed heterozygous (*Oxtr*^+/−^) and homozygous *Oxtr* (*Oxtr*^−/−^) conditional knockout mice (Fig. 1B). Consistently, a dual-probe FISH revealed that the majority of *Oxtr* mRNA-positive cells were *Amigo2* mRNA-expressing cells and the number of *Oxtr*-positive cells in the CA2 of *Oxtr*^−/−^ mice was markedly reduced compared to that of WT mice (Fig. 1C), confirming the efficiency of Cre-loxP-mediated deletion of CA2 *Oxtr*. However, histological analysis by cresyl violet staining revealed that *Oxtr* deletion did not significantly affect the total number of neurons in CA2 stratum pyramidale compared to WT littermates (WT: *n* = 3; *Oxtr*^−/−^: *n* = 4; *t*_(5)_ = 0.19, *P* = 0.85; unpaired Student’s *t*-test; Fig. 1D).

### Conditional deletion of CA2 *Oxtr* impairs the persistence of long-term SRM

**Fig. 2:**
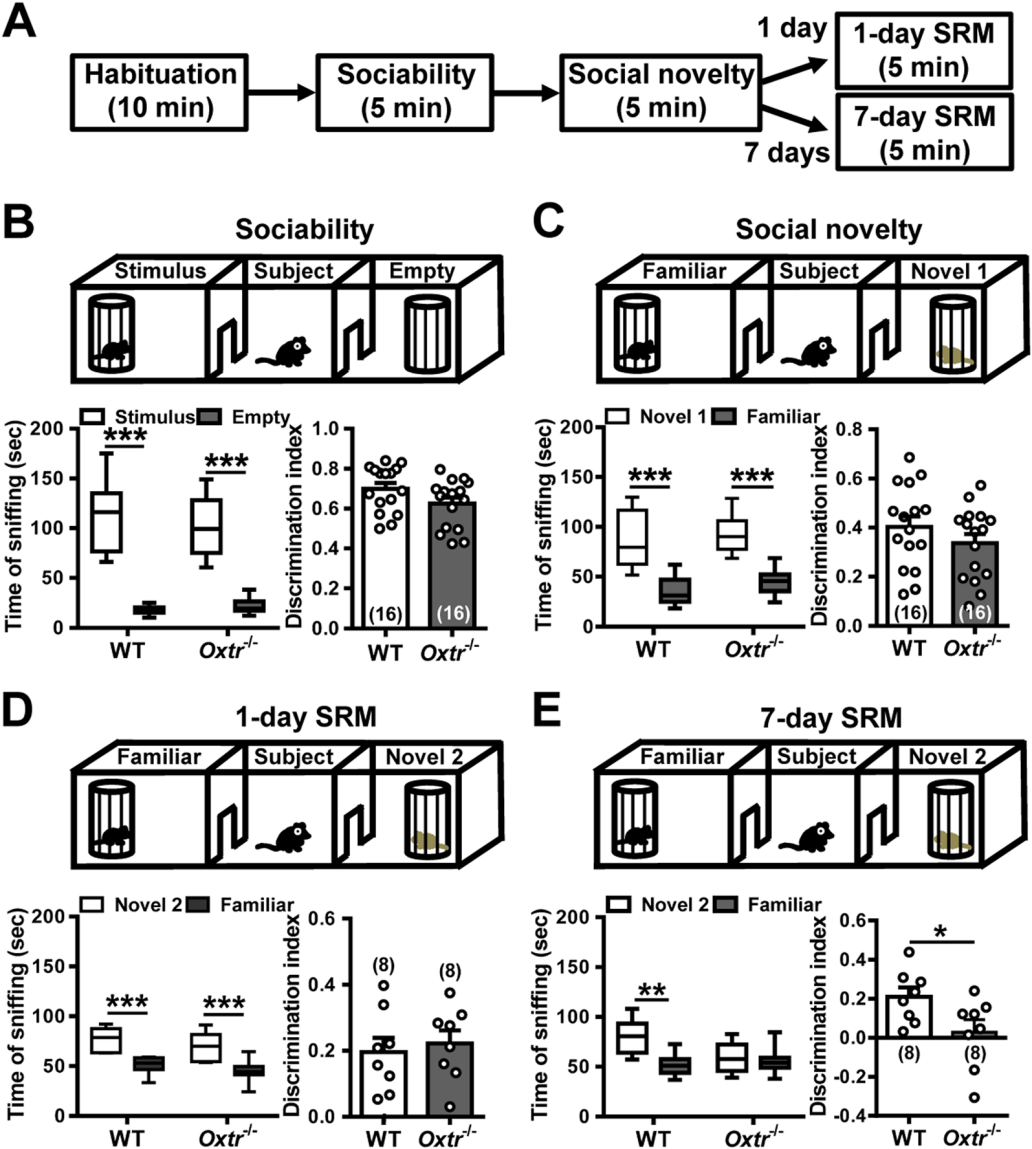
Deletion of *Oxtr* in CA2 excitatory neurons leads to impaired persistence long-term SRM. **A** Schematic representation of the experimental design. **B** Top, schematic representation of the three-chamber sociability test. Bottom left, time spent by the subject mouse in sniffing directed at a wire cage containing the juvenile stimulus mouse or an empty wire cage. Both WT and *Oxtr*^−/−^ subject mice spent significantly more time interacting with the wire cage containing the juvenile stimulus mouse than the empty wire cage. Bottom right, discrimination index (stimulus minus empty) was similar between WT and *Oxtr*^−/−^ subject mice in the sociability test. **C** Top, schematic representation of the three-chamber social novelty preference test. Bottom left, time spent by the subject mouse in sniffing directed at the wire cage containing a familiar mouse or a novel 1 mouse, 10 min after the sociability test. Both WT and *Oxtr*^−/−^ subject mice spent significantly more time sniffing the cage containing the novel mouse than the familiar mouse. Bottom right, discrimination index (novel 1 minus familiar) was comparable between WT and *Oxtr*^−/−^ subject mice in the social novelty preference test. **D** Top, schematic representation of the three-chamber long-term SRM test. Bottom left, time spent by the subject mouse in sniffing directed at the wire cage containing a familiar mouse or a novel 2 mouse, 1 day after the initial interaction. Both WT and *Oxtr*^−/−^ subject mice spent significantly more time sniffing the cage containing the novel mouse than the familiar mouse. Bottom right, discrimination index (novel 2 minus familiar) was similar between WT and *Oxtr*^−/−^ subject mice in 1-day long-term SRM test. **E** Top, schematic representation of the three-chamber long-term SRM test. Bottom, time spent by the subject mouse in sniffing directed at the wire cage containing a familiar mouse or a novel 2 mouse, 7 days after the initial interaction. WT, but not *Oxtr*^−/−^, subject mice spent significantly more time sniffing the cage containing the novel mouse than the familiar mouse. Bottom right, discrimination index (novel 2 minus familiar) of *Oxtr*^−/−^ subject mice was significantly less than WT subject mice in 7-day long-term SRM test. 1-day and 7-day long-term SRM tests were performed on independent groups. The total number of animal examined is indicated by *n* in parenthesis. Error bars represent the SEM; *P < 0.05, **P < 0.01, ***P < 0.001

To determine the impact of CA2 OXTR deletion on SRM persistence, we used a three-chamber paradigm (Fig. 2A), a task widely used for studying social approach behaviors in mice [7, 32]. We initially compared the performance of WT and *Oxtr*^−/−^ mice in sociability assay, which examines the subject mouse’s preference for interacting with a social stimulus (a unfamiliar mouse enclosed in a wire cage) over a non-social one (an empty wire cage). We found no difference between WT and *Oxtr*^−/−^ mice in preference for the wire cage containing the stimulus mouse. Discrimination index was similar between WT (*n* = 16) and *Oxtr*^−/−^ mice (*n* = 16) in sociability test (*t*_(30)_ = 1.78, *P* = 0.09; unpaired Student’s *t*-test; Fig. 2B). We next performed the social novelty preference test that examines the ability of the subject mouse to discriminate between novel and familiar social stimuli. Following the sociability test, a novel stimulus mouse was presented in the previously empty wire cage. As shown in Fig. 2C, both WT (*n* = 16) and *Oxtr*^−/−^ mice (*n* = 16) exhibited a more extensive investigation of the novel mouse compared to the familiar mouse, with no difference between the genotypes. Discrimination index was comparable between WT and *Oxtr*^−/−^ mice in social novelty preference test (*t*_(30)_ = 1.18, *P* = 0.25; unpaired Student’s *t*-test). When mice were tested SRM 1 day after the training session (1-day long-term SRM), both WT (*n* = 8) and *Oxtr*^−/−^ mice (*n* = 8) revealed intact memory retention. They both displayed a significant and similar preference for spending more time in exploring the novel mouse than the familiar mouse. There was no significant differences between WT and *Oxtr*^−/−^ mice in discrimination index of 1-day long-term SRM test (*t*_(14)_ = 0.45, *P* = 0.66; unpaired Student’s *t*-test; Fig. 2D). In contrast, when tested SRM 7 days after the initial interaction (7-day long-term SRM), we found that *Oxtr*^−/−^ mice (*n* = 8) were unable to discriminate between novel and familiar mouse as they spent equal time investigating both the novel and familiar mouse (*t*_(7)_ = 0.45, *P* = 0.66; paired Student’s *t*-test), indicating the degree of 7-day long-term SRM was impaired by *Oxtr* deletion. WT mice (*n* = 8), however, spent significantly more time exploring the novel mouse than the familiar mouse (*t*_(7)_ = 4.05 *P* = 0.005; paired Student’s *t*-test). As a consequence, a statistically significant discrimination index was observed between WT and *Oxtr*^−/−^ mice in 7-day long-term SRM (*t*_(14)_ = 2.27, *P* = 0.039; unpaired Student’s *t*-test; Fig. 2E). Together, these results suggest that CA2 OXTRs is crucial for the persistence of long-term SRM, but not for sociability, social novelty preference and 1-day SRM.

### Targeted activation of PVN OXT neurons promotes the persistence of long-term SRM

**Fig. 3:**
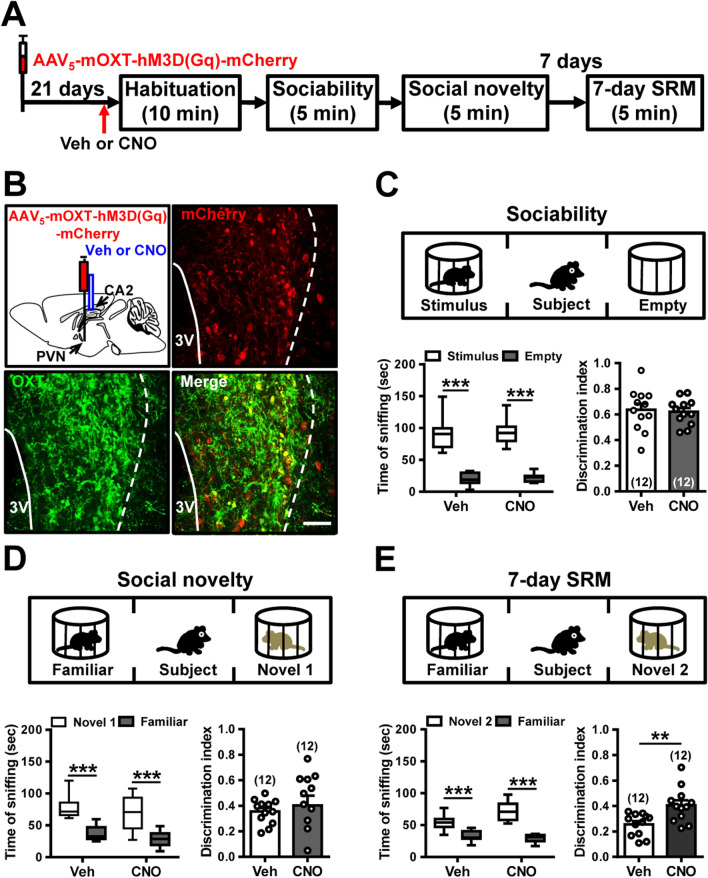
Activation of PVN OXT neurons enhance long-term SRM. **A** Schematic representation of the experimental design. **B** Schematic representation of viral injection and CNO administration. Three weeks after stereotaxic injection of AAV_5_-mOXT-hM3D(Gq)-mCherry into the PVN, mice were subjected to three-chamber paradigm test and long-term SRM retention was tested 7 days after the initial interaction. Mice were bilaterally injected with either vehicle (Veh) or CNO into dCA2 20 min before the initial interaction. Representative images showing the co-expression of hM3D(Gq)-mCherry and OXT immunoreactivity in the PVN. Scale bar represents 50 μm. **C** Top, schematic representation of the three-chamber sociability test. Bottom left, time spent by the subject mouse in sniffing directed at the wire cage containing the juvenile stimulus mouse or the empty wire cage. hM3D(Gq)/Veh and hM3D(Gq)/CNO subject mice spent significantly more time interacting with the wire cage containing the juvenile stimulus mouse than the empty wire cage. Bottom right, discrimination index (stimulus minus empty) was similar among groups in the sociability test. **D** Top, schematic representation of the three-chamber social novelty preference test. Bottom left, time spent by the subject mouse in sniffing directed at the wire cage containing a familiar mouse or a novel 1 mouse, 10 min after the sociability test. hM3D(Gq)/Veh and hM3D(Gq)/CNO subject mice spent significantly more time sniffing the cage containing the novel mouse than the familiar mouse. Bottom right, discrimination index (novel 1 minus familiar) was comparable among groups in the social novelty preference test. **E** Top, schematic representation of the three-chamber long-term SRM test. Bottom, time spent by the subject mouse in sniffing directed at the wire cage containing a familiar mouse or a novel 2 mouse, 7 days after the initial interaction. hM3D(Gq)/Veh and hM3D(Gq)/CNO subject mice spent significantly more time sniffing the cage containing the novel mouse than the familiar mouse. Bottom right, discrimination index (novel 2 minus familiar) of hM3D(Gq)/CNO subject mice was significantly higher than those of hM3D(Gq)/Veh subject mice in 7-day long-term SRM test. The total number of animal examined is indicated by *n* in parenthesis. Error bars represent the SEM; **P < 0.01, *** *P* < 0.001

We and others previously reported that OXT neurons in the PVN send axonal projections to dCA2 [12, 30, 33]. To determine if the endogenous OXT system participates in regulating the formation of long-term SRM, we used chemogenetic approach to activate PVN OXT neurons during SRM encoding. We bilaterally injected the PVN with an AAV_5_ vector expressing hM3D(Gq)-mCherry under the control of the endogenous mouse OXT (mOXT) promoter [AAV_5_-mOXT-hM3D(Gq)-mCherry] (Fig. 3A). Three weeks after viral injection, mice were subjected to bilateral injection of either vehicle or CNO into dCA2 and then subjected to the three-chamber paradigm test 20 min after vehicle or CNO injection. We verified the neuronal specificity of viral expression by imaging mCherry, whose expression was confined to neurons co-expressing OXT in the PVN (Fig. 3B). We found that acute in vivo activation of the PVN OXT neurons with CNO (*n* = 12) did not affect sociability (*t*_(22)_ = 0.28, *P* = 0.78; unpaired Student’s *t*-test; Fig. 3C) or social novelty preference (*t*_(22)_ = 0.57, *P* = 0.58; unpaired Student’s *t*-test; Fig. 3D) compared with vehicle treatment group (*n* = 12). Although no differences between groups were present in sociability and social novelty preference tests, acute activation of the PVN OXT neurons (hM3D(Gq)/CNO group) caused a significant increase in the amount of 7-day long-term SRM. Discrimination index was significantly higher in the hM3D(Gq)/CNO-treated group (*n* = 12) than in the hM3D(Gq)/vehicle-treated group (*n* = 12; *t*_(22)_ = 3.19, *P* = 0.0043; unpaired Student’s *t*-test; Fig. 3E).

**Fig. 4:**
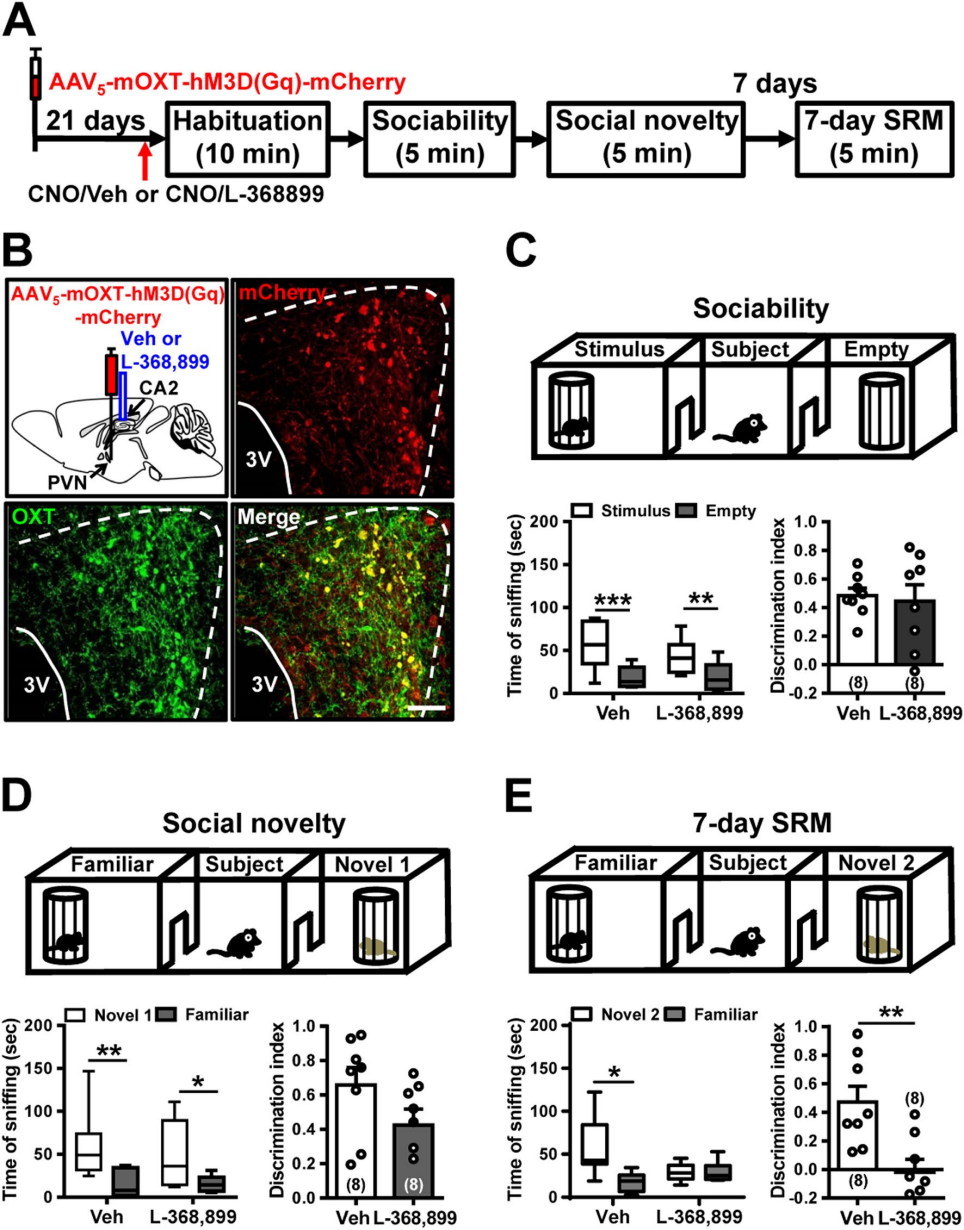
Pharmacological antagonism of OXTR in dCA2 blocks chemogenetic enhancement of long-term SRM. **A** Schematic representation of the experimental design. **B** Schematic representation of viral injection and CNO administration. Three weeks after stereotaxic injection of AAV_5_-mOXT-hM3D(Gq)-mCherry into the PVN, mice were subjected to three-chamber paradigm test and long-term SRM retention was tested 7 days after the initial interaction. Mice were bilaterally administered of vehicle (Veh) or an OXTR antagonist L-368,899 into dCA2 10 min before CNO injection, followed by three-chamber paradigm test 20 min after CNO injection. Representative images showing the co-expression of hM3D(Gq)-mCherry and OXT immunoreactivity in the PVN. mCherry signals of axonal projections were observed in dCA2. Scale bar represents 50 μm. **C** Top, schematic representation of the three-chamber sociability test. Bottom left, time spent by the subject mouse in sniffing directed at the wire cage containing the juvenile stimulus mouse or the empty wire cage. hM3D(Gq)/CNO/Veh and hM3D(Gq)/CNO/L-368,899 subject mice spent significantly more time interacting with the wire cage containing the juvenile stimulus mouse than the empty wire cage. Bottom right, discrimination index (stimulus minus empty) was similar between groups in the sociability test. **D** Top, schematic representation of the three-chamber social novelty preference test. Bottom left, time spent by the subject mouse in sniffing directed at the wire cage containing a familiar mouse or a novel 1 mouse, 10 min after the sociability test. hM3D(Gq)/CNO/Veh and hM3D(Gq)/CNO/L-368,899 subject mice spent significantly more time sniffing the cage containing the novel mouse than the familiar mouse. Bottom right, discrimination index (novel 1 minus familiar) was comparable between groups in the social novelty preference test. **E** Top, schematic representation of the three-chamber long-term SRM test. Bottom, time spent by the subject mouse in sniffing directed at the wire cage containing a familiar mouse or a novel 2 mouse, 7 days after the initial interaction. hM3D(Gq)/CNO/Veh, but not hM3D(Gq)/CNO/L-368,899, subject mice spent significantly more time sniffing the cage containing the novel mouse than the familiar mouse. Bottom right, discrimination index (novel 2 minus familiar) of hM3D(Gq)/CNO/Veh subject mice was significantly higher than that of hM3D(Gq)/CNO/L-368,899 subject mice in 7-day long-term SRM test. The total number of animal examined is indicated by *n* in parenthesis. Error bars represent the SEM; *P < 0.05, **P < 0.01, ***P < 0.001

To better characterize the involvement of locally released OXT and subsequent activation of OXTR-mediated signaling in dCA2 during SRM encoding, we bilaterally administered vehicle or an OXTR antagonist L-368,899 (12 μg in PBS, 0.5 μl) into dCA2 of hM3D(Gq)-mCherry-expressed mice 10 min before CNO injection, followed by three-chamber paradigm test 20 min after CNO injection (Fig. 4A). Post hoc histological examination of brain sections revealed robust co-expression of mCherry with OXT in the PVN (Fig. 4B). In comparison with vehicle-treated mice, we found no significant effect of L-368,899 treatment in sociability (*t*_(14)_ = 0.31, *P* = 0.76; unpaired Student’s *t*-test; Fig. 4C) or social novelty preference (*t*_(14)_ = 1.69, *P* = 0.11; unpaired Student’s *t*-test; Fig. 4D) in hM3D(Gq)/CNO mice. However, pretreatment with L-368,899 blocked the enhancement of 7-day long-term SRM seen in hM3D(Gq)-mCherry-expressed mice following CNO treatment. Post hoc analyses indicated that the enhancement of discrimination ratio seen with activation of the PVN OXT neurons was abolished when OXTRs were blocked (*t*_(14)_ = 3.43, *P* = 0.004; unpaired Student’s *t*-test; Fig. 4E). These results suggest that increasing endogenous hypothalamic OXT secretion in dCA2 can promote the persistence of long-term SRM.

### dCA2 activity is essential for long-term SRM formation

**Fig. 5:**
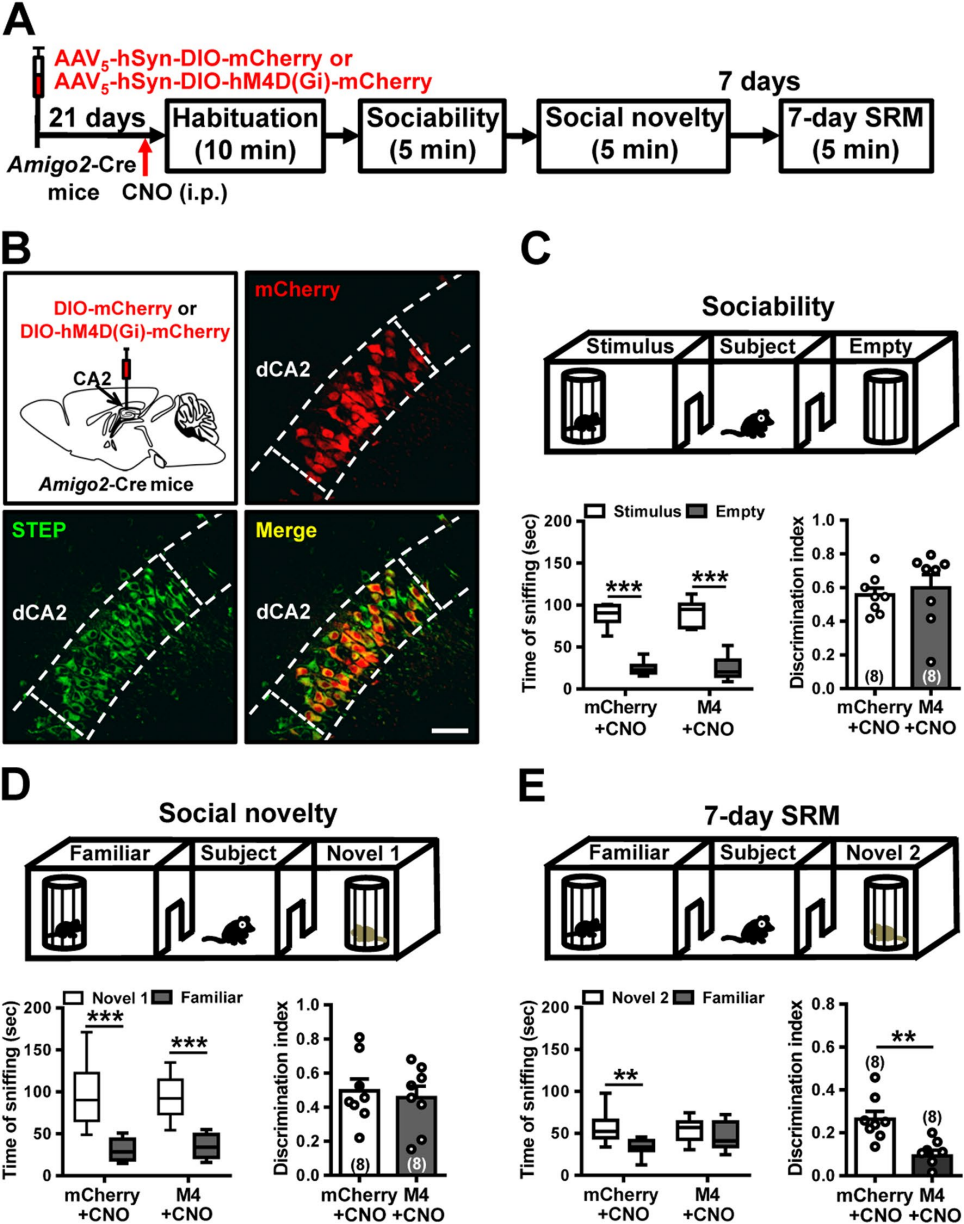
dCA2 neuronal activity is necessary for the persistence of long-term SRM. **A** Schematic representation of the experimental design. **B** Schematic representation of viral injection. Three weeks after stereotaxic injection of AAV_5_-hSyn-DIO-hM4D(Gi)-mCherry or AAV_5_-hSyn-DIO-mCherry into the dCA2 of *Amigo2*-Cre mice, mice were subjected to three-chamber paradigm test and long-term SRM retention was tested 7 days after the initial interaction. Mice were intraperitoneally injected with CNO 30 min before the initial interaction. Representative images showing the co-expression of hM4D(Gi)-mCherry and STEP immunoreactivity in dCA2 pyramidal neurons. Scale bar represents 50 μm. **C** Top, schematic representation of the three-chamber sociability test. Bottom left, time spent by the subject mouse in sniffing directed at the wire cage containing the juvenile stimulus mouse or the empty wire cage. Both hM4D(Gi)/CNO and mCherry/CNO subject mice spent significantly more time interacting with the wire cage containing the juvenile stimulus mouse than the empty wire cage. Bottom right, discrimination index (stimulus minus empty) was similar between groups in the sociability test. **D** Top, schematic representation of the three-chamber social novelty preference test. Bottom left, time spent by the subject mouse in sniffing directed at the wire cage containing a familiar mouse or a novel 1 mouse, 10 min after the sociability test. Both hM4D(Gi)/CNO and mCherry/CNO subject mice spent significantly more time sniffing the cage containing the novel mouse than the familiar mouse. Bottom right, discrimination index (novel 1 minus familiar) was comparable between groups in the social novelty preference test. **E** Top, schematic representation of the three-chamber long-term SRM test. Bottom, time spent by the subject mouse in sniffing directed at the wire cage containing a familiar mouse or a novel 2 mouse, 7 days after the initial interaction. mCherry/CNO, but not hM4D(Gi)/CNO, subject mice spent significantly more time sniffing the cage containing the novel mouse than the familiar mouse. Bottom right, discrimination index (novel 2 minus familiar) of hM4D(Gi)/CNO subject mice was significantly less than mCherry/CNO subject mice in 7-day long-term SRM test. The total number of animal examined is indicated by *n* in parenthesis. Error bars represent the SEM; **P < 0.01, ***P < 0.001

To probe causality between dCA2 activity and the formation of long-term SRM, we targeted dCA2 pyramidal neurons for chemogenetic silencing during SRM encoding. We compared the effects of intraperitoneal injection of CNO in *Amigo2*-Cre mice infected in dCA2 with AAV_5_-hSyn-DIO-hM4D(Gi)-mCherry or control AAV_5_-hSyn-DIO-mCherry. Three weeks after viral infection, all mice underwent three-chamber paradigm test 30 min after CNO injection (5 mg/kg) (Fig. 5A). Post hoc histological examination of brain sections revealed robust and bilateral co-expression of mCherry with STEP in dCA2 pyramidal neurons (Fig. 5B). Both groups exhibited significant preference for the wire cage containing the stimulus mouse than the empty wire cage in sociability test (Fig. 5C) and the novel mouse than the familiar mouse in social novelty preference test (Fig. 5D). We found no main effects in discrimination index of sociability (mCherry + CNO: *n* = 8; hM4D(Gi) + CNO: *n* = 8; *t*_(14)_ = 0.49, *P* = 0.64; unpaired Student’s *t*-test; Fig. 5C) and social novelty preference (mCherry + CNO: *n* = 8; hM4D(Gi) + CNO: *n* = 8; *t*_(14)_ = 0.42, *P* = 0.68; unpaired Student's *t*-test; Fig. 5D) between groups. However, we observed a significant effect in 7-day long-term SRM between groups. Post hoc analysis indicated that discrimination index was significantly less for DIO-hM4D(Gi)-mCherry-expressed mice (*n* = 8) than control mCherry-expressed mice (*n* = 8; *t*_(14)_ = 3.82, *P* = 0.002; unpaired Student’s *t*-test; Fig. 5E).

### A selective dCA2-to-vCA1 circuit involves in long-term SRM formation

**Fig. 6:**
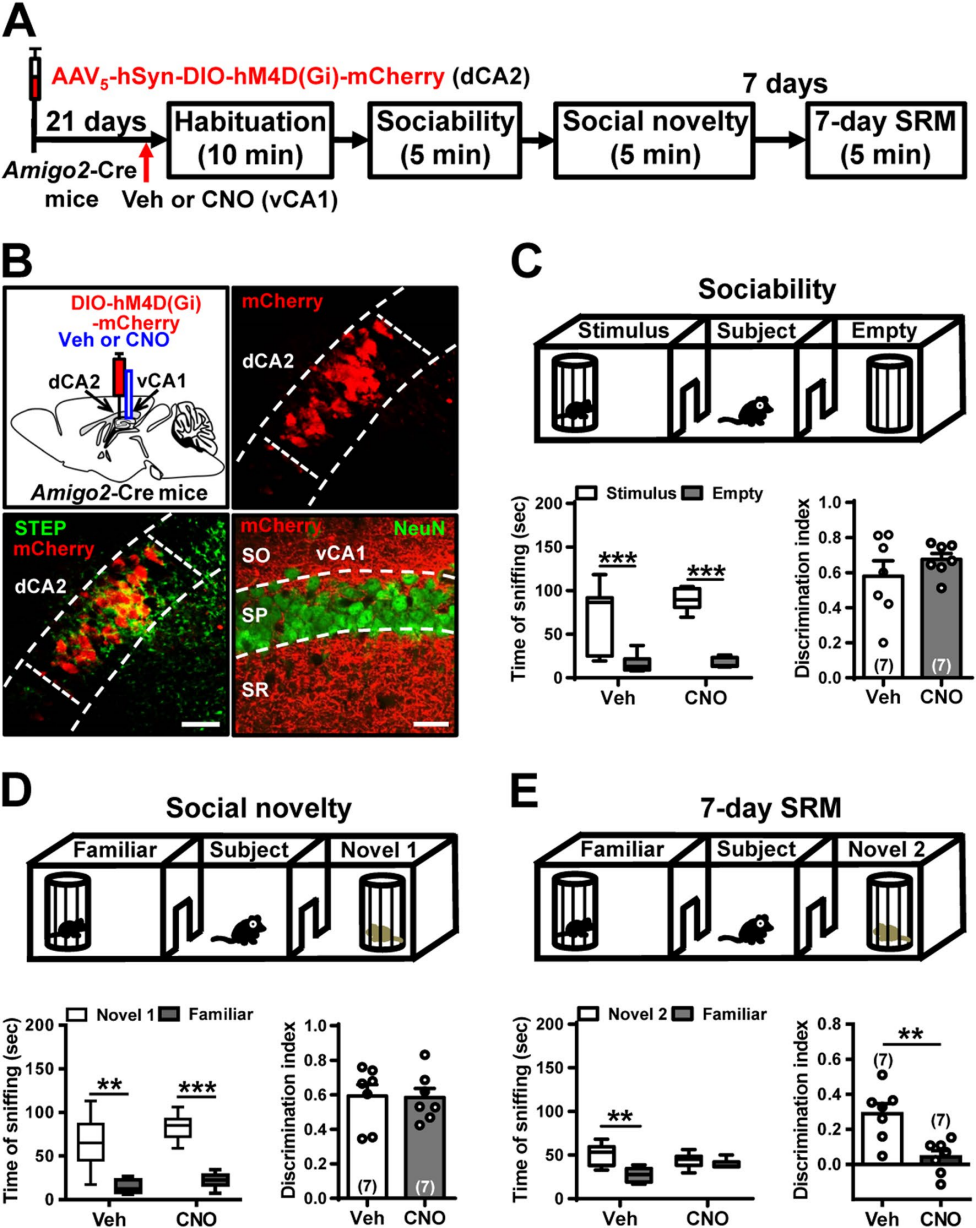
dCA2 projections to vCA1 is necessary for long-term SRM. **A** Schematic representation of the experimental design. **B** Schematic representation of viral injection and CNO administration. Three weeks after stereotaxic injection of AAV_5_-hSyn-DIO-hM4D(Gi)-mCherry into the dCA2 of *Amigo2*-Cre mice, mice were subjected to three-chamber paradigm test and long-term SRM retention was tested 7 days after the initial interaction. Mice were bilaterally administered of vehicle (Veh) or CNO into vCA1 20 min before the initial interaction. Representative images showing the co-expression of hM4D(Gi)-mCherry and STEP immunoreactivity in dCA2 pyramidal neurons. Scale bars represent 50 μm. mCherry signals of axonal projections were observed in vCA1. Scale bar represents 100 μm. *NeuN* neuronal nuclear protein, *SO* stratum oriens, *SP* stratum pyramidale, *SR* stratum radiatum. **C** Top, schematic representation of the three-chamber sociability test. Bottom left, time spent by the subject mouse in sniffing directed at the wire cage containing the juvenile stimulus mouse or the empty wire cage. Both hM4D(Gi)/Veh and hM4D(Gi)/CNO subject mice spent significantly more time interacting with the wire cage containing the juvenile stimulus mouse than the empty wire cage. Bottom right, discrimination index (stimulus minus empty) was similar between hM4D(Gi)/Veh and hM4D(Gi)/CNO subject mice in the sociability test. **D** Top, schematic representation of the three-chamber social novelty preference test. Bottom left, time spent by the subject mouse in sniffing directed at the wire cage containing a familiar mouse or a novel 1 mouse, 10 min after the sociability test. Both hM4D(Gi)/Veh and hM4D(Gi)/CNO subject mice spent significantly more time sniffing the cage containing the novel mouse than the familiar mouse. Bottom right, discrimination index (novel 1 minus familiar) was comparable between hM4D(Gi)/Veh and hM4D(Gi)/CNO subject mice in the social novelty preference test. **E** Top, schematic representation of the three-chamber long-term SRM test. Bottom, time spent by the subject mouse in sniffing directed at the wire cage containing a familiar mouse or a novel 2 mouse, 7 days after the initial interaction. Both hM4D(Gi)/Veh and hM4D(Gi)/CNO subject mice spent significantly more time sniffing the cage containing the novel mouse than the familiar mouse. Bottom right, discrimination index (novel 2 minus familiar) of hM4D(Gi)/CNO subject mice was significantly less than hM4D(Gi)/Veh subject mice in 7-day long-term SRM test. The total number of animal examined is indicated by *n* in parenthesis. Error bars represent the SEM; **P < 0.01, ***P < 0.001

To explore dCA2 outputs necessary for controlling long-term SRM formation, we focused on vCA1 and dLS that have been identified as downstream targets of dCA2 and potential neuroanatomical substrates involved in social and emotional behaviors [13, 31]. To interrogate the role of dCA2-to-vCA1 projections in long-term SRM, we performed bilateral injections of AAV_5_-hSyn-DIO-hM4D(Gi)-mCherry into dCA2 of *Amigo2*-Cre mice. After allowing 2 weeks for viral expression, mice were implanted with cannula targeting vCA1. One week later, mice underwent three-chamber paradigm test. Twenty minutes before sociability test, mice were injected with vehicle or CNO via cannula targeting vCA1 (Fig. 6A). Post hoc histological examination of brain sections revealed robust co-expression of mCherry with STEP in dCA2 (Fig. 6B, bottom left panel). We also observed dense projection fibers from dCA2 to vCA1 (Fig. 6B, bottom right panel). In comparison with vehicle-treated mice (*n* = 7), we found no significant effect of CNO treatment (*n* = 7) in discrimination index of sociability (*t*_(12)_ = 1.02, *P* = 0.33; unpaired Student’s *t*-test; Fig. 6C) or social novelty preference (*t*_(12)_ = 0.10, *P* = 0.92; unpaired Student’s *t*-test; Fig. 6D) in hM4D(Gi)-mCherry-expressed mice. However, a statistically significant difference was observed between vehicle- (*n* = 7) and CNO-treated hM4D(Gi)-mCherry-expressed mice (*n* = 7) in discrimination index of 7-day long-term SRM (*t*_(12)_ = 3.66, *P* = 0.003; unpaired Student’s *t*-test; Fig. 6E).

**Fig. 7:**
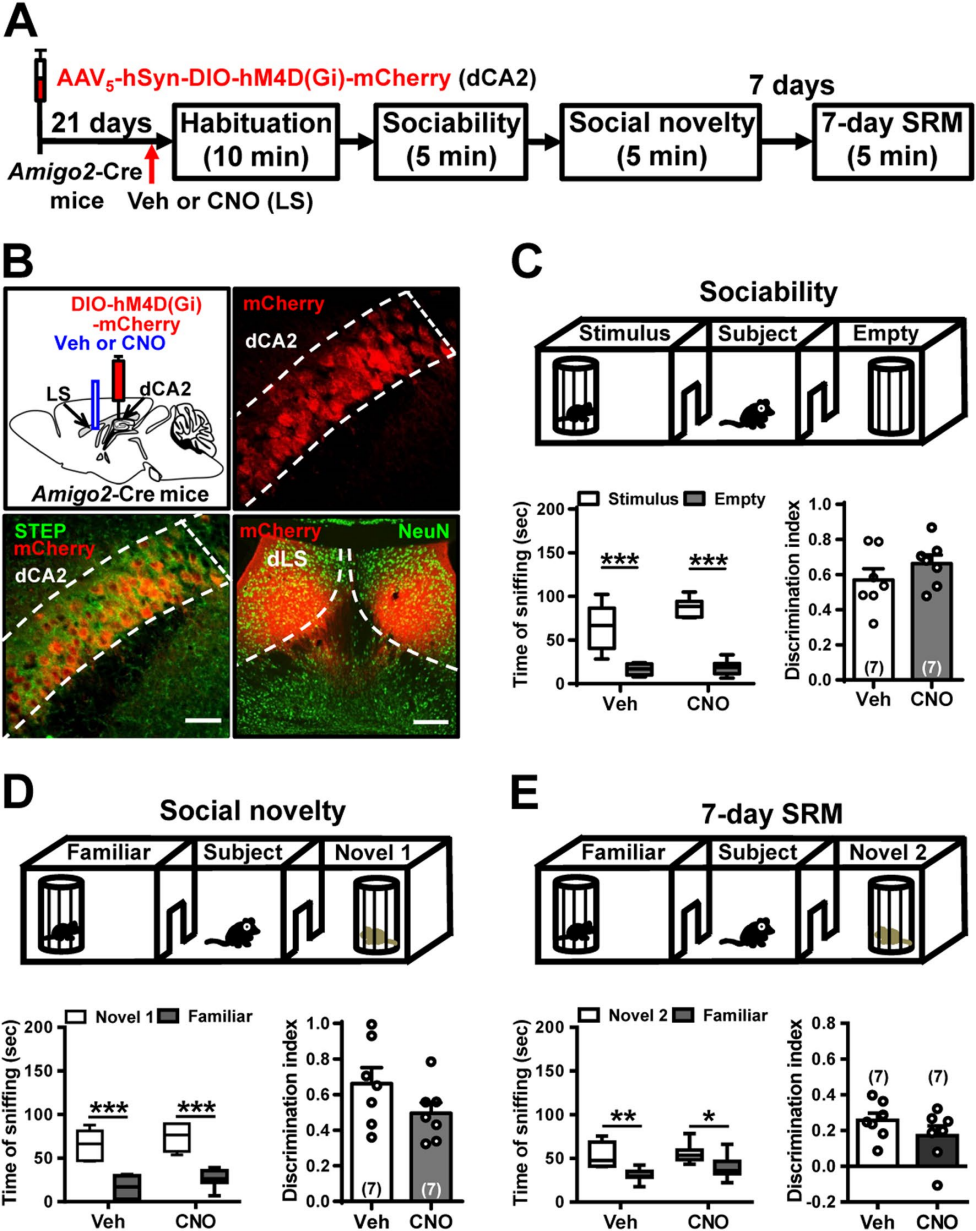
dCA2 projections to dLS is not involved in long-term SRM. **A** Schematic representation of the experimental design. **B** Schematic representation of viral injection. Three weeks after stereotaxic injection of AAV_5_-hSyn-DIO-hM4D(Gi)-mCherry into the dCA2 of *Amigo2*-Cre mice, mice were subjected to three-chamber paradigm test and long-term SRM retention was assessed 7 days after the initial interaction. Mice were bilaterally administered of vehicle (Veh) or CNO into LS 20 min before the initial interaction. Representative images showing the co-expression of hM4D(Gi)-mCherry and STEP immunoreactivity in dCA2 pyramidal neurons. Scale bars represent 50 μm. mCherry signals of axonal projections were observed in dLS. Scale bar represents 100 μm. *NeuN* neuronal nuclear protein. **C** Top, schematic representation of the three-chamber sociability test. Bottom left, time spent by the subject mouse in sniffing directed at the wire cage containing the juvenile stimulus mouse or the empty wire cage. Both hM4D(Gi)/Veh and hM4D(Gi)/CNO subject mice spent significantly more time interacting with the wire cage containing the juvenile stimulus mouse than the empty wire cage. Bottom right, discrimination index (stimulus minus empty) was similar between hM4D(Gi)/Veh and hM4D(Gi)/CNO subject mice in the sociability test. **D** Top, schematic representation of the three-chamber social novelty preference test. Bottom left, time spent by the subject mouse in sniffing directed at the wire cage containing a familiar mouse or a novel 1 mouse, 10 min after the sociability test. Both hM4D(Gi)/Veh and hM4D(Gi)/CNO subject mice spent significantly more time sniffing the cage containing the novel mouse than the familiar mouse. Bottom right, discrimination index (novel 1 minus familiar) was comparable between hM4D(Gi)/Veh and hM4D(Gi)/CNO subject mice in the social novelty preference test. **E** Top, schematic representation of the three-chamber long-term SRM test. Bottom, time spent by the subject mouse in sniffing directed at the wire cage containing a familiar mouse or a novel 2 mouse, 7 days after the initial interaction. Both hM4D(Gi)/Veh and hM4D(Gi)/CNO subject mice spent significantly more time sniffing the cage containing the novel mouse than the familiar mouse. Bottom right, discrimination index (novel 2 minus familiar) was similar between hM4D(Gi)/Veh and hM4D(Gi)/CNO subject mice in 7-day long-term SRM test. The total number of animal examined is indicated by *n* in parenthesis. Error bars represent the SEM; *P < 0.05, **P < 0.01, ***P < 0.001

To establish whether dCA2 promotes long-term SRM through its outputs to dLS, we bilaterally injected AAV_5_-hSyn-DIO-hM4D(Gi)-mCherry into dCA2 of *Amigo2*-Cre mice. After allowing 2 weeks for viral expression, mice were implanted with cannula targeting dLS. One week later, mice underwent three-chamber paradigm test. Twenty minutes before sociability test, mice were injected with vehicle or CNO via cannula targeting dLS (Fig. 7A). Post hoc histological examination of brain sections revealed robust co-expression of mCherry with STEP in dCA2 (Fig. 7B, bottom left panel). We also observed dense projection fibers from dCA2 to dLS (Fig. 7B, bottom right panel). We found that CNO-treated hM4D(Gi)-mCherry-expressed mice (*n* = 7) performed similarly to vehicle-treated hM4D(Gi)-mCherry-expressed mice (*n* = 7) in discrimination index of sociability (*t*_(12)_ = 1.15, *P* = 0.27; unpaired Student’s *t*-test; Fig. 7C), social novelty preference (*t*_(12)_ = 1.54, *P* = 0.15; unpaired Student’s *t*-test; Fig. 7D) and 7-day long-term SRM (*t*_(12)_ = 1.27, *P* = 0.23; unpaired Student’s *t*-test; Fig. 7E).

### Activation of dCA2-to-vCA1 projections ameliorates long-term SRM deficit in *Oxtr*^−/−^ mice

**Fig. 8:**
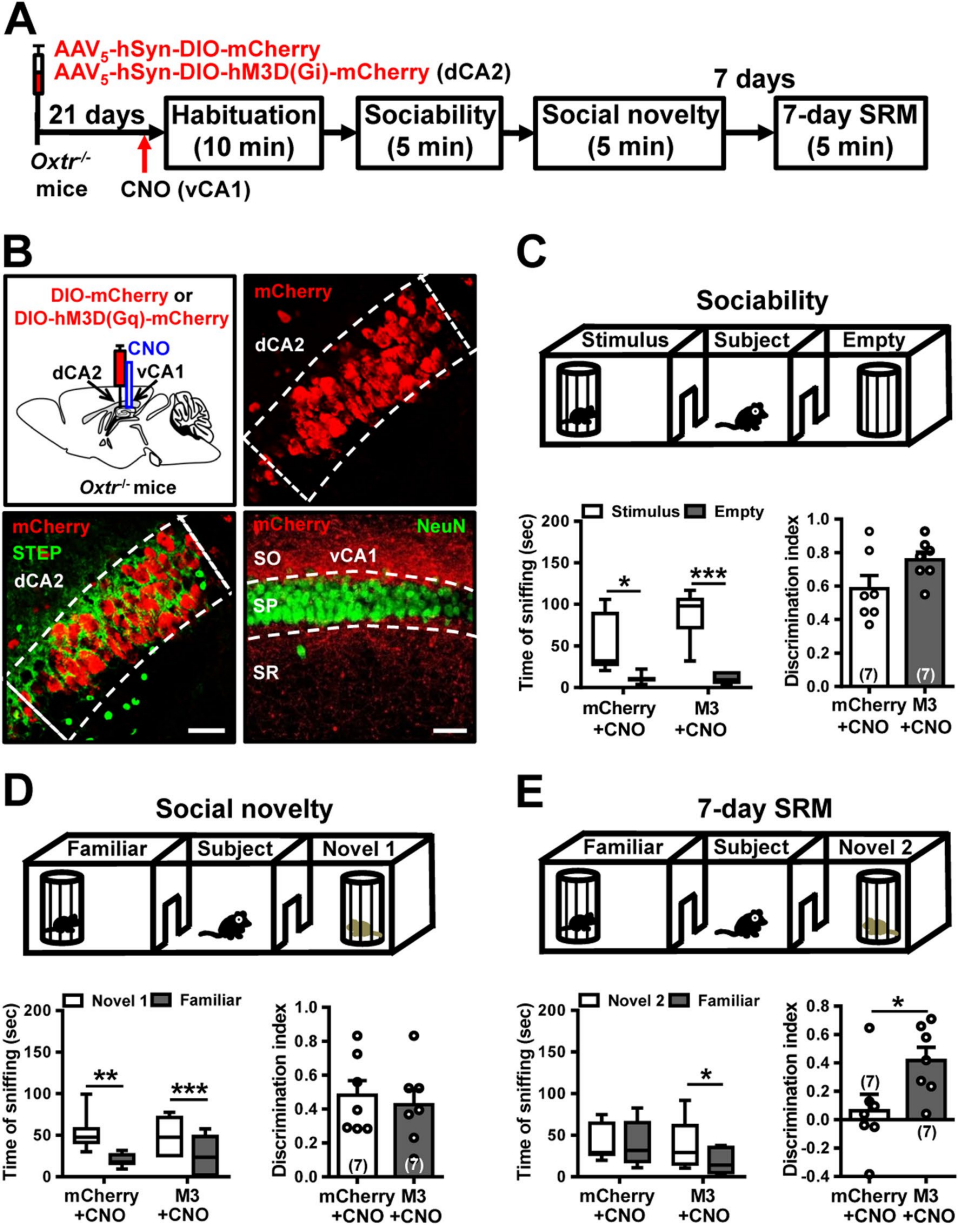
Activation of dCA2-to-vCA1 projections rescues long-term SRM deficit in *Oxtr*^−/−^ mice. **A** Schematic representation of the experimental design. **B** Schematic representation of viral injection and CNO administration. Three weeks after stereotaxic injection of AAV_5_-hSyn-DIO-hM3D(Gq)-mCherry or AAV_5_-hSyn-DIO-mCherry into the dCA2 of *Oxtr*^−/−^ mice, mice were subjected to three-chamber paradigm test and long-term SRM retention was tested 7 days after the initial interaction. *Oxtr*^−/−^ mice were bilaterally administered of CNO into vCA1 20 min before the initial interaction. Representative images showing the co-expression of hM3D(Gq)-mCherry and STEP immunoreactivity in dCA2 pyramidal neurons of *Oxtr*^−/−^ mice. Scale bar represents 50 μm. mCherry signals of axonal projections were observed in vCA1. Scale bar represents 100 μm. *NeuN* neuronal nuclear protein, *SO* stratum oriens, *SP* stratum pyramidale, *SR* stratum radiatum. **C** Top, schematic representation of the three-chamber sociability test. Bottom left, time spent by the subject mouse in sniffing directed at the wire cage containing the juvenile stimulus mouse or the empty wire cage. Both mCherry/CNO and hM3D(Gq)/CNO *Oxtr*^−/−^ mice spent significantly more time interacting with the wire cage containing the juvenile stimulus mouse than the empty wire cage. Bottom right, discrimination index (stimulus minus empty) were similar between mCherry/CNO and hM3D(Gq)/CNO *Oxtr*^−/−^ mice in the sociability test. **D** Top, schematic representation of the three-chamber social novelty preference test. Bottom left, time spent by the subject mouse in sniffing directed at the wire cage containing a familiar mouse or a novel 1 mouse, 10 min after the sociability test. Both mCherry/CNO and hM3D(Gq)/CNO *Oxtr*^−/−^ mice spent significantly more time sniffing the cage containing the novel mouse than the familiar mouse. Bottom right, discrimination index (novel 1 minus familiar) was comparable between mCherry/CNO and hM3D(Gq)/CNO *Oxtr*^−/−^ mice in the social novelty preference test. **E** Top, schematic representation of the three-chamber long-term SRM test. Bottom, time spent by the subject mouse in sniffing directed at the wire cage containing a familiar mouse or a novel 2 mouse, 7 days after the initial interaction. hM3D(Gq)/CNO, but not mCherry/CNO, *Oxtr*^−/−^ mice spent significantly more time sniffing the cage containing the novel mouse than the familiar mouse. Bottom right, discrimination index (novel 2 minus familiar) of hM3D(Gq)/CNO *Oxtr*^−/−^ mice was significantly higher than mCherry/CNO *Oxtr*^−/−^ mice in 7-day long-term SRM test. The total number of animal examined is indicated by *n* in parenthesis. Error bars represent the SEM; *P < 0.05, **P < 0.01, ***P < 0.001

Activation of OXTRs in the CA2 is known to increase neuronal excitability [34]. To investigate whether specific aspect of long-term SRM deficit in *Oxtr*^−/−^ mice is indeed due to deficits in CA2 function within the dCA2-to-vCA1 circuit, we examined if the selective chemogenetic activation of the dCA2-to-vCA1 circuit could rescue long-term SRM deficit. To do this, we performed bilateral injections of control AAV_5_-hSyn-DIO-mCherry or AAV_5_-hSyn-DIO-hM3D(Gq)-mCherry into dCA2 of *Oxtr*^−/−^ mice. After allowing 2 weeks for viral expression, mice were implanted with cannula targeting vCA1. One week later, mice underwent three-chamber paradigm test. Twenty minutes before sociability test, mice were injected with CNO via cannula targeting vCA1 (Fig. 8A). Post hoc histological examination of brain sections revealed robust co-expression of mCherry with STEP in dCA2 (Fig. 8B, bottom left panel). We also observed dense projection fibers from dCA2 to vCA1 (Fig. 8B, bottom right panel). The hM3D(Gq)-mCherry-expressed *Oxtr*^−/−^ mice (*n* = 7) performed similarly to mCherry-expressed *Oxtr*^−/−^ mice (*n* = 7) in discrimination index of sociability (*t*_(12)_ = 1.89, *P* = 0.08; unpaired Student’s *t*-test; Fig. 8C) and social novelty preference (*t*_(12)_ = 0.45, *P* = 0.66; unpaired Student’s *t*-test; Fig. 8D). In 7-day long-term SRM test, a statistically significant discrimination index was observed between mCherry-expressed and hM3D(Gq)-mCherry-expressed *Oxtr*^−/−^ mice in 7-day long-term SRM (*t*_(12)_ = 2.37, *P* = 0.03; unpaired Student’s *t*-test; Fig. 8E). Together, these findings suggest that dCA2 promotes the persistence of long-term SRM, at least in part, through its projections to vCA1. Remarkably, activation of the dCA2-to-vCA1 circuit can effectively ameliorate long-term SRM deficit in *Oxtr*^−/−^ mice.

## Discussion

SRM is considered a hippocampus-dependent memory and can persist over long periods of time following successful encoding [7, 35]. In this study, using three-chamber paradigm test to measure long-term SRM in *Amigo2*-Cre mediated *Oxtr*^−/−^ mice, we show that endogenous OXTR signaling in dCA2 plays an essential role in regulating the persistence of long-term SRM. Through chemogenetic terminal-specific manipulations, we identify the contribution of dCA2 and its projections to vCA1 in the formation of long-term SRM. More importantly, targeted activation of dCA2-to-vCA1 projections effectively ameliorates long-term SRM deficit observed in *Oxtr*^−/−^ mice. Collectively, these findings expand on previous studies linking dCA2 function to SRM [9, 13–15] and support the notion that CA2 OXTR signaling is crucial for the long-term persistence of SRM [12]. To the best of our knowledge, this is the first study aiming to uncover the functional role of the dCA2-to-vCA1 circuit in mediating endogenous OXTergic modulation of long-term SRM formation.

Rodents can retain SRM for at least 1 week [36, 37]. In contrast, SRM persists no more than a few hours in socially isolated animals [15, 38]. These findings strongly indicate that there exist group housing-related factors prolonging an hour-long labile SRM to days. The potential contributors are neuromodulators, which are employed in SRM formation [35, 39]. The hippocampal CA2 region has been proven to be a crucial hub in SRM processing [9, 13]. A host of neuromodulatory inputs have been suggested to converge on the CA2 subfield [40, 41], among which OXT and its closely related neuropeptide arginine vasopressin have been received much more attention. We have previously reported that conditional deletion of CA2/CA3a OXTR impairs the persistence of long-term SRM [12], but no consensus has thus far emerged. However, Chiang and coworkers [22] have shown that silencing dCA3 had no significant effect on SRM. Because the CA2 heavily innervates the CA3a [28], this anatomical connectivity might bias interpretation of the role of CA3a OXTRs in governing long-term SRM. Unfortunately, the Cre/loxP conditional knockout strategy used in our previous study did not reach selective deletion of *Oxtr* from CA2 or CA3a neurons. This shortcoming can be overcome with the aid of *Amigo2*-Cre mice, in which Cre expression is largely restricted to CA2 pyramidal neurons [9]. Although one previous study showed expression of *Amigo2* mRNA not only in the CA2 but also in some CA3a cells in mouse brain [42], we found this rare. In the present study, by taking advantage of this transgenic mouse line that enables precise deletion of CA2 *Oxtr*, we focused our research on the regulatory role of endogenous OXTR signaling in dCA2 and its outputs in controlling long-term SRM persistence. In support, we clearly demonstrate that mice lacking CA2 OXTRs have a deficit in the persistence of long-term SRM and, in particular, chemogenetic activation of OXT neurons in the PVN enhances long-term SRM, an effect that was blocked by local application of an OXTR antagonist L-368,899 in dCA2. In addition, we find no changes in sociability and social novelty preference in *Oxtr*^−/−^ mice, suggesting a specific role for dCA2 OXTR signaling in long-term SRM. Consistent with our previous work [12], we show that memory retention was unaffected at 1 day after encoding but significantly impaired at 7 days after the initial interaction in mice lacking CA2 *Oxtr*, supporting that OXTR signaling in the CA2 is specifically critical for the persistence of long-term SRM rather than short-term SRM. We, therefore, speculate that OXTR signaling in dCA2 during SRM encoding may strengthen the salience of social information and enable this information to be consolidated into a long-term form, dissociating decay of memory traces with the passage of time. It is known that long-term SRM is dependent on protein synthesis and cAMP responsive element binding protein activity [7]; thus, it seems likely that OXTR signaling might exert effects via similar cellular mechanisms to promote the persistence of long-term SRM, which needs to be investigated in the future.

There is also evidence that local pharmacological antagonism of the vasopressin (AVP) 1b receptors in the CA2 prevents extended SRM induced by optogenetic stimulation of PVN AVP projection neurons, suggesting that CA2 AVP1b receptor signaling is critical for social memory formation [37]. It has been hypothesized that OXT and AVP play differential roles in the sequential phases of SRM. OXT appears to be critical for the acquisition rather than the consolidation phase of memory and AVP appears to be important for SRM consolidation [4, 43, 44]. Further studies are required to address this possibility.

Our findings reinforce the importance of dCA2 neuronal activity in long-term SRM formation. Indeed, we demonstrate that chemogenetic silencing of dCA2 pyramidal neurons impairs long-term SRM. This finding aligns with a previous study demonstrating that excitotoxic *N*-methyl-d-aspartate lesions of the CA2 impair SRM in mice [14]. Similarly, Hitti and Siegelbaum [9] showed that chronic genetically engineered shutdown of CA2 pyramidal cell synaptic transmission with tetanus neurotoxin also results in an impairment of SRM without affecting sociability. Furthermore, acute chemogenetic or optogenetic silencing dCA2 impaired SRM processing in *Amigo2*-Cre mice infected in dCA2 with AAV_2_-hSyn-DIO-hM4D(Gi)-mCitrine or AAV_2/5_-EF1α-DIO-eArch3.0-eYFP, respectively [13]. A pressing question that follows these observations is how dCA2 contributes to SRM formation. In rodents, SRM is mediated mainly by olfactory cues perceived via the olfactory system [45]. Considering that the CA2 receives non-spatial information from the entorhinal cortex [30, 46, 47] and the entorhinal cortex receives extensive inputs from the olfactory bulb and piriform cortex, both areas that devote to processing of olfactory information [48], it is therefore possible that the CA2 may contribute to SRM by incorporating these social and olfactory inputs to create a memory trace necessary for the recognition process [39].

Our characterization of an excitatory pathway from dCA2 to vCA1 may account for endogenous OXTergic modulation of long-term SRM formation. The dynamic activity of this projection has been implicated in processing of SRM [13]. While dCA2 also sends anatomical projections to dLS, which was recently shown to participate in promoting social aggression [31], we did not observe any effect of terminal-specific silencing of dCA2-to-dLS projections on long-term SRM. More importantly, we provide evidence that acute activation of the dCA2-to-vCA1 circuit can effectively improve long-term SRM deficit observed in *Oxtr*^−/−^ mice. Thus, interplay within OXTR signaling-modulated dCA2-to-vCA1 circuit likely plays a specific role in long-term SRM formation. Notably, a previous study has shown that vCA1 and its projections to the NAc shell play a necessary and sufficient role in storing SRM [15], raising an intriguing possibility that dCA2 targets vCA1 pyramidal neurons that project to the NAc shell to subserve long-term SRM storage. Additional studies are needed to clarify this issue.

Our study has some limitations. First, this study only used male mice for test subjects. Therefore, additional studies are warranted to determine whether our findings can be extrapolated to female mice. Although the evidence remains inconclusive, there are significant differences in sociability and social recognition between male and female mice [25, 26]. Such differences could be associated with sex differences in circulating gonadal hormones. Second, we only used the three-chamber paradigm test for evaluating long-term SRM. In addition to the classical three-chamber paradigm test, the social habituation–dishabituation paradigm and the social discrimination paradigm test were used to evaluate SRM [49]. Further studies will need to determine whether our findings can be extrapolated to other behavioral paradigm tests.

## Conclusions

Taken together, our results integrate CA2 OXTR signaling into the dCA2-to-vCA1 circuit to promote the persistence of long-term SRM. Our findings contribute to a better understanding of neuromodulatory and neural circuit mechanisms contributing to long-term SRM formation. Relevant information may provide a novel therapeutic avenue to the treatment of social cognitive disabilities seen in autism spectrum disorders and other psychiatric disorders [50–52].

## Supplementary Information

**Additional file 1: **Summary of statistical analyses and values for the current study.

## Data Availability

The authors confirm that all data generated and analyzed during this study are either included in this published article or available from the corresponding authors upon reasonable request.

## Abbreviations

AAV: Adeno-associated virus
AAV2: Adeno-associated virus type 2
AAV5: Adeno-associated virus type 5
AP: Anteroposterior
CA3a: Cornu ammonis area 3a
cAMP: Cyclic adenosine monophosphate
CNO: Clozapine-*N*-oxide
DAPI: 4′,6-Diamidino-2-phenylindole
dCA2: Dorsal Cornu ammonis area 2
DIO: Double-inverted orientation
dLS: Dorsal lateral septum
DMSO: Dimethyl sulfoxide
DV: Dorsoventral
FISH: Fluorescent in situ hybridization
hM3D(Gq): Human M3 muscarinic receptor coupled to Gq
hM4D(Gi): Human M4 muscarinic receptor coupled to Gi
hSyn: Human synapsin 1
L-368,899: (2S)-2-Amino-*N*-[(1S,2S,4R)-7,7-dimethyl-1-[[[4-(2-methylphenyl)-1-piperazinyl]sulfonyl]methyl]bicyclo[2.2.1]hept-2-yl]-4-(methylsulfonyl)butanamide
LS: Lateral septum
mOXT: Mouse oxytocin
ML: Mediolateral
NAc: Nucleus accumbens
OXT: Oxytocin
OXTR: Oxytocin receptor
*Oxtr*^J/J^: Homozygous *Oxtr*-floxed
*Oxtr*^Venus^^−^^Neo/+^: Heterozygous OXTR-Venus knock-in
PBS: Phosphate-buffered saline
PFA: Paraformaldehyde
PVN: Paraventricular nucleus
SEM: Standard error of the mean
SRM: Social recognition memory
STEP: Striatum-enriched protein-tyrosine phosphatase
TBS-T: Tris-buffered saline with Tween 20
vCA1: Ventral Cornu ammonis area 1
WT: Wild-type

## Availability of data and materials

All data generated or analyzed during this study are included in the published article.
