# Supplementary material for: A dorsal CA2 to ventral CA1 circuit contributes to oxytocinergic modulation of long-term social recognition memory
Source: J Biomed Sci. 2022 Jul 10;29:50. doi: 10.1186/s12929-022-00834-x (PMC9272559; doi:10.1186/s12929-022-00834-x)
Supplement: Supplementary file 1 — Additional file 1: Summary of statistical analyses and values for the current study. [file 12929_2022_834_MOESM1_ESM.docx]

**Summary of statistical analyses and values for the current study.**

|  | **TEST USED** | | ***n*** | | | **Descriptive STATS(AVERAGE, VARIANCE)** | | **P VALUE** | | **DEGREES OF FREEDOM & F/T/r/etc VALUE** | |
| --- | --- | --- | --- | --- | --- | --- | --- | --- | --- | --- | --- |
| **FIGURE NUMBER** | **WHICH TEST** | **PAGE** | **VALUE** | **DEFINED** | **PAGE** | **REPORTED** | **PAGE** | **VALUE** | **PAGE** | **DEFINED** | **PAGE** |
| 1D right panel | Two-tailed unpaired student’s t-test | Legend fig 1 | WT, n = 3; Oxtr^-/-^, n = 4 | Total number of mice per group | Legend fig 1 | Error bar are mean ± SEM | Legend fig 1 | p = 0.8546 | Legend fig 1 | t = 0.1929; df = 5;  R square = 0.0074 | Legend fig 1 |
| 2A left panel | Two-tailed paired student’s t-test | Legend fig 2 | WT, n = 16 | Total number of mice per group | Legend fig 2 | Error bar are mean ± SEM | Legend fig 2 | p < 0.0001 | Legend fig 2 | t = 10.59; df = 15;  R square = 0.88 | Legend fig 2 |
|  |  |  | Oxtr-/-, n = 16 |  |  |  |  | p < 0.0001 |  | t = 10.87; df = 15;  R square = 0.89 |  |
| 2A right panel | Two-tailed unpaired student’s t-test | Legend fig 2 | WT, n = 16; Oxtr-/-, n = 16 | Total number of mice per group | Legend fig 2 | Error bar are mean ± SEM | Legend fig 2 | p = 0.0856 | Legend fig 2 | t = 1.778; df = 30;  R square = 0.0095 | Legend fig 2 |
| 2B left panel | Two-tailed paired student’s t-test | Legend fig 2 | WT, n = 16 | Total number of mice per group | Legend fig 2 | Error bar are mean ± SEM | Legend fig 2 | p < 0.0001 | Legend fig 2 | t =7.493; df = 15;  R square = 0.79 | Legend fig 2 |
|  |  |  | Oxtr-/-, n = 16 |  |  |  |  | p < 0.0001 |  | t = 8.346; df = 15;  R square = 0.82 |  |
| 2B right panel | Two-tailed unpaired student’s t-test | Legend fig 2 | WT, n = 16; Oxtr-/-, n = 16 | Total number of mice per group | Legend fig 2 | Error bar are mean ± SEM | Legend fig 2 | p = 0.2484 | Legend fig 2 | t = 1.177; df = 30;  R square = 0.044 | Legend fig 2 |
| 2C left panel | Two-tailed paired student’s t-test | Legend fig 2 | WT, n = 8 | Total number of mice per group | Legend fig 2 | Error bar are mean ± SEM | Legend fig 2 | p = 0.0025 | Legend fig 2 | t = 4.608; df = 7;  R square = 0.75 | Legend fig 2 |
|  |  |  | Oxtr-/-, n = 8 |  |  |  |  | p = 0.0010 |  | t = 5.390; df = 7;  R square = 0.81 |  |
| 2C right panel | Two-tailed unpaired student’s t-test | Legend fig 2 | WT, n = 8; Oxtr-/-, n = 8 | Total number of mice per group | Legend fig 2 | Error bar are mean ± SEM | Legend fig 2 | p = 0.6577 | Legend fig 2 | t = 0.4527; df = 14;  R square = 0.014 | Legend fig 2 |
| 2D left panel | Two-tailed paired student’s t-test | Legend fig 2 | WT, n = 8 | Total number of mice per group | Legend fig 2 | Error bar are mean ± SEM | Legend fig 2 | p = 0.0049 | Legend fig 2 | t = 4.050; df = 7;  R square = 0.70 | Legend fig 2 |
|  |  |  | Oxtr-/-, n = 8 |  |  |  |  | p = 0.66 |  | t = 0.4542; df = 7;  R square = 0.029 |  |
| 2D right panel | Two-tailed unpaired student’s t-test | Legend fig 2 | WT, n = 8; Oxtr-/-, n = 8 | Total number of mice per group | Legend fig 2 | Error bar are mean ± SEM | Legend fig 2 | p = 0.039 | Legend fig 2 | t = 2.273; df = 14;  R square = 0.27 | Legend fig 2 |
| 3B left panel | Two-tailed paired student’s t-test | Legend fig 3 | Veh, n = 12 | Total number of mice per group | Legend fig 3 | Error bar are mean ± SEM | Legend fig 3 | p < 0.0001 | Legend fig 3 | t = 9.411; df = 11;  R square = 0.89 | Legend fig 3 |
|  |  |  | CNO, n = 12 |  |  |  |  | p < 0.0001 |  | t = 11.73; df = 11;  R square = 0.93 |  |
| 3B right panel | Two-tailed unpaired student’s t-test | Legend fig 3 | Veh, n = 12; CNO, n = 12 | Total number of mice per group | Legend fig 3 | Error bar are mean ± SEM | Legend fig 3 | p = 0.7788 | Legend fig 3 | t = 0.2844; df = 22;  R square = 0.0037 | Legend fig 3 |
| 3C left panel | Two-tailed paired student’s t-test | Legend fig 3 | Veh, n = 12 | Total number of mice per group | Legend fig 3 | Error bar are mean ± SEM | Legend fig 3 | p < 0.0001 | Legend fig 3 | t = 10.59; df = 11;  R square = 0.91 | Legend fig 3 |
|  |  |  | CNO, n = 12 |  |  |  |  | p = 0.0002 |  | t = 5.382; df = 11;  R square = 0.72 |  |
| 3C right panel | Two-tailed unpaired student’s t-test | Legend fig 3 | Veh, n = 12; CNO, n = 12 | Total number of mice per group | Legend fig 3 | Error bar are mean ± SEM | Legend fig 3 | p = 0.5757 | Legend fig 3 | t = 0.5682; df = 22;  R square = 0.014 | Legend fig 3 |
| 3D left panel | Two-tailed paired student’s t-test | Legend fig 3 | Veh, n = 12 | Total number of mice per group | Legend fig 3 | Error bar are mean ± SEM | Legend fig 3 | p < 0.0001 | Legend fig 3 | t = 9.207; df = 11;  R square = 0.89 | Legend fig 3 |
|  |  |  | CNO, n = 12 |  |  |  |  | p < 0.0001 |  | t = 8.262; df = 11;  R square = 0.86 |  |
| 3D right panel | Two-tailed unpaired student’s t-test | Legend fig 3 | Veh, n = 12; CNO, n = 12 | Total number of mice per group | Legend fig 3 | Error bar are mean ± SEM | Legend fig 3 | p = 0.0043 | Legend fig 3 | t = 3.185; df = 22;  R square = 0.3156 | Legend fig 3 |
| 4B left panel | Two-tailed paired student’s t-test | Legend fig 4 | Veh, n = 8 | Total number of mice per group | Legend fig 4 | Error bar are mean ± SEM | Legend fig 4 | p = 0.0006 | Legend fig 4 | t = 5.957; df = 7;  R square = 0.84 | Legend fig 4 |
|  |  |  | L-398,899, n = 8 |  |  |  |  | p = 0.0052 |  | t = 4.004; df = 7;  R square = 0.70 |  |
| 4B right panel | Two-tailed unpaired student’s t-test | Legend fig 4 | Veh, n = 8;  L-398,899, n = 8 | Total number of mice per group | Legend fig 4 | Error bar are mean ± SEM | Legend fig 4 | p = 0.7598 | Legend fig 4 | t = 0.3118; df = 14;  R square = 0.0069 | Legend fig 4 |
| 4C left panel | Two-tailed paired student’s t-test | Legend fig 4 | Veh, n = 8 | Total number of mice per group | Legend fig 4 | Error bar are mean ± SEM | Legend fig 4 | p = 0.0065 | Legend fig 4 | t = 3.825; df = 7;  R square = 0.84 | Legend fig 4 |
|  |  |  | L-398,899, n = 8 |  |  |  |  | p = 0.0281 |  | t = 2.760; df = 7;  R square = 0.52 |  |
| 4C right panel | Two-tailed unpaired student’s t-test | Legend fig 4 | Veh, n = 8;  L-398,899, n = 8 | Total number of mice per group | Legend fig 4 | Error bar are mean ± SEM | Legend fig 4 | p = 0.1124 | Legend fig 4 | t = 1.694; df = 14;  R square = 0.17 | Legend fig 4 |
| 4D left panel | Two-tailed paired student’s t-test | Legend fig 4 | Veh, n = 8 | Total number of mice per group | Legend fig 4 | Error bar are mean ± SEM | Legend fig 4 | p = 0.0207 | Legend fig 4 | t = 2.973; df = 7;  R square = 0.56 | Legend fig 4 |
|  |  |  | L-398,899, n = 8 |  |  |  |  | p = 0.8612 |  | t = 0.1813; df = 7;  R square = 0.0047 |  |
| 4D right panel | Two-tailed unpaired student’s t-test | Legend fig 4 | Veh, n = 8;  L-398,899, n = 8 | Total number of mice per group | Legend fig 4 | Error bar are mean ± SEM | Legend fig 4 | p = 0.0041 | Legend fig 4 | t = 3.430; df = 14;  R square = 0.46 | Legend fig 4 |
| 5B left panel | Two-tailed paired student’s t-test | Legend fig 5 | mCherry+CNO, n = 8 | Total number of mice per group | Legend fig 5 | Error bar are mean ± SEM | Legend fig 5 | p < 0.0001 | Legend fig 5 | t = 16.32; df = 7;  R square = 0.97 | Legend fig 5 |
|  |  |  | M4+CNO, n = 8 |  |  |  |  | p < 0.0001 |  | t = 11.50; df = 7;  R square = 0.95 |  |
| 5B right panel | Two-tailed unpaired student’s t-test | Legend fig 5 | mCherry+CNO, n = 8; M4+CNO, n = 8 | Total number of mice per group | Legend fig 5 | Error bar are mean ± SEM | Legend fig 5 | p = 0.6350 | Legend fig 5 | t = 0.4852; df = 14;  R square = 0.01654 | Legend fig 5 |
| 5C left panel | Two-tailed paired student’s t-test | Legend fig 5 | mCherry+CNO, n = 8 | Total number of mice per group | Legend fig 5 | Error bar are mean ± SEM | Legend fig 5 | p = 0.0029 | Legend fig 5 | t = 4.480; df = 7;  R square = 0.74 | Legend fig 5 |
|  |  |  | M4+CNO, n = 8 |  |  |  |  | p = 0.0007 |  | t = 5.693; df = 7;  R square = 0.82 |  |
| 5C right panel | Two-tailed unpaired student’s t-test | Legend fig 5 | mCherry+CNO, n = 8; M4+CNO, n = 8 | Total number of mice per group | Legend fig 5 | Error bar are mean ± SEM | Legend fig 5 | p = 0.6830 | Legend fig 5 | t = 0.4170; df = 14;  R square = 0.01227 | Legend fig 5 |
| 5D left panel | Two-tailed paired student’s t-test | Legend fig 5 | mCherry+CNO, n = 8 | Total number of mice per group | Legend fig 5 | Error bar are mean ± SEM | Legend fig 5 | p = 0.0011 | Legend fig 5 | t = 5.300; df = 7;  R square = 0.97 | Legend fig 5 |
|  |  |  | M4+CNO, n = 8 |  |  |  |  | p = 0.0193 |  | t = 3.022; df = 7;  R square = 0.57 |  |
| 5D right panel | Two-tailed unpaired student’s t-test | Legend fig 5 | mCherry+CNO, n = 8; M4+CNO, n = 8 | Total number of mice per group | Legend fig 5 | Error bar are mean ± SEM | Legend fig 5 | p = 0.0019 | Legend fig 5 | t = 3.821; df = 14;  R square = 0.51 | Legend fig 5 |
| 6B left panel | Two-tailed paired student’s t-test | Legend fig 6 | Veh, n = 7 | Total number of mice per group | Legend fig 6 | Error bar are mean ± SEM | Legend fig 6 | p = 0.0048 | Legend fig 6 | t = 4.356; df = 6;  R square = 0.7547 | Legend fig 6 |
|  |  |  | CNO, n = 7 |  |  |  |  | p < 0.0001 |  | t = 14.42; df = 6;  R square = 0.97 |  |
| 6B right panel | Two-tailed unpaired student’s t-test | Legend fig 6 | Veh, n = 7; CNO, n = 7 | Total number of mice per group | Legend fig 6 | Error bar are mean ± SEM | Legend fig 6 | p = 0.3297 | Legend fig 6 | t = 1.016; df = 12;  R square = 0.079 | Legend fig 6 |
| 6C left panel | Two-tailed paired student’s t-test | Legend fig 6 | Veh, n = 7 | Total number of mice per group | Legend fig 6 | Error bar are mean ± SEM | Legend fig 6 | p = 0.0027 | Legend fig 6 | t = 4.887; df = 6;  R square = 0.80 | Legend fig 6 |
|  |  |  | CNO, n = 7 |  |  |  |  | p < 0.0001 |  | t = 10.32; df = 6;  R square = 0.95 |  |
| 6C right panel | Two-tailed unpaired student’s t-test | Legend fig 6 | Veh, n = 7; CNO, n = 7 | Total number of mice per group | Legend fig 6 | Error bar are mean ± SEM | Legend fig 6 | p = 0.9208 | Legend fig 6 | t = 0.1015; df = 12;  R square = 0.00086 | Legend fig 6 |
| 6D left panel | Two-tailed paired student’s t-test | Legend fig 6 | Veh, n = 7 | Total number of mice per group | Legend fig 6 | Error bar are mean ± SEM | Legend fig 6 | p = 0.0036 | Legend fig 6 | t = 4.622; df = 6;  R square = 0.78 | Legend fig 6 |
|  |  |  | CNO, n = 7 |  |  |  |  | p = 0.2282 |  | t = 1.342; df = 6;  R square = 0.23 |  |
| 6D right panel | Two-tailed unpaired student’s t-test | Legend fig 6 | Veh, n = 7; CNO, n = 7 | Total number of mice per group | Legend fig 6 | Error bar are mean ± SEM | Legend fig 6 | p = 0.0033 | Legend fig 6 | t = 3.658; df = 12;  R square = 0.53 | Legend fig 6 |
| 7B left panel | Two-tailed paired student’s t-test | Legend fig 7 | Veh, n = 7 | Total number of mice per group | Legend fig 7 | Error bar are mean ± SEM | Legend fig 7 | p = 0.0029 | Legend fig 7 | t = 4.835; df = 6;  R square = 0.80 | Legend fig 7 |
|  |  |  | CNO, n = 7 |  |  |  |  | p < 0.0001 |  | t = 15.14; df = 6;  R square = 0.97 |  |
| 7B right panel | Two-tailed unpaired student’s t-test | Legend fig 7 | Veh, n = 7; CNO, n = 7 | Total number of mice per group | Legend fig 7 | Error bar are mean ± SEM | Legend fig 7 | p = 0.2708 | Legend fig 7 | t = 1.154; df = 12;  R square = 0.10 | Legend fig 7 |
| 7C left panel | Two-tailed paired student’s t-test | Legend fig 7 | Veh, n = 7 | Total number of mice per group | Legend fig 7 | Error bar are mean ± SEM | Legend fig 7 | p = 0.0002 | Legend fig 7 | t = 8.271; df = 6;  R square = 0.92 | Legend fig 7 |
|  |  |  | CNO, n = 7 |  |  |  |  | p < 0.0001 |  | t = 10.27; df = 6;  R square = 0.95 |  |
| 7C right panel | Two-tailed unpaired student’s t-test | Legend fig 7 | Veh, n = 7; CNO, n = 7 | Total number of mice per group | Legend fig 7 | Error bar are mean ± SEM | Legend fig 7 | p = 0.1497 | Legend fig 7 | t = 1.539; df = 12;  R square = 0.16 | Legend fig 7 |
| 7D left panel | Two-tailed paired student’s t-test | Legend fig 7 | Veh, n = 7 | Total number of mice per group | Legend fig 7 | Error bar are mean ± SEM | Legend fig 7 | p = 0.0017 | Legend fig 7 | t = 5.367; df = 6;  R square = 0.83 | Legend fig 7 |
|  |  |  | CNO, n = 7 |  |  |  |  | p = 0.0331 |  | t = 2.754; df = 6;  R square = 0.56 |  |
| 7D right panel | Two-tailed unpaired student’s t-test | Legend fig 7 | Veh, n = 7; CNO, n = 7 | Total number of mice per group | Legend fig 7 | Error bar are mean ± SEM | Legend fig 7 | p = 0.2268 | Legend fig 7 | t = 1.274; df = 12;  R square = 0.12 | Legend fig 7 |
| 8B left panel | Two-tailed paired student’s t-test | Legend fig 8 | mCherry+CNO, n = 7 | Total number of mice per group | Legend fig 8 | Error bar are mean ± SEM | Legend fig 8 | p = 0.0187 | Legend fig 8 | t = 3.197; df = 6;  R square = 0.63 | Legend fig 8 |
|  |  |  | M3+CNO, n = 7 |  |  |  |  | p = 0.0004 |  | t = 7.238; df = 6;  R square = 0.90 |  |
| 8B right panel | Two-tailed unpaired student’s t-test | Legend fig 8 | mCherry+CNO, n = 7; M3+CNO, n = 7 | Total number of mice per group | Legend fig 8 | Error bar are mean ± SEM | Legend fig 8 | p = 0.0830 | Legend fig 8 | t = 1.891; df = 12;  R square = 0.23 | Legend fig 8 |
| 8C left panel | Two-tailed paired student’s t-test | Legend fig 8 | mCherry+CNO, n = 7 | Total number of mice per group | Legend fig 8 | Error bar are mean ± SEM | Legend fig 8 | p = 0.0076 | Legend fig 8 | t = 3.941; df = 6;  R square = 0.72 | Legend fig 8 |
|  |  |  | M3+CNO, n = 7 |  |  |  |  | p < 0.0001 |  | t = 11.99; df = 6;  R square = 0.96 |  |
| 8C right panel | Two-tailed unpaired student’s t-test | Legend fig 8 | mCherry+CNO, n = 7; M3+CNO, n = 7 | Total number of mice per group | Legend fig 8 | Error bar are mean ± SEM | Legend fig 8 | p = 0.6585 | Legend fig 8 | t = 0.4532; df = 12;  R square = 0.017 | Legend fig 8 |
| 8D left panel | Two-tailed paired student’s t-test | Legend fig 8 | mCherry+CNO, n = 7 | Total number of mice per group | Legend fig 8 | Error bar are mean ± SEM | Legend fig 8 | p = 0.8515 | Legend fig 8 | t = 0.1954; df = 6;  R square = 0.0063 | Legend fig 8 |
|  |  |  | M3+CNO, n = 7 |  |  |  |  | p = 0.0177 |  | t = 3.241; df = 6;  R square = 0.64 |  |
| 8D right panel | Two-tailed unpaired student’s t-test | Legend fig 8 | mCherry+CNO, n = 7; M3+CNO, n = 7 | Total number of mice per group | Legend fig 8 | Error bar are mean ± SEM | Legend fig 8 | p = 0.0353 | Legend fig 8 | t = 2.371; df = 12;  R square = 0.3191 | Legend fig 8 |
